# Phylogeographic structure and ecological niche modelling reveal signals of isolation and postglacial colonisation in the European stag beetle

**DOI:** 10.1371/journal.pone.0215860

**Published:** 2019-04-25

**Authors:** Karen Cox, Niall McKeown, Gloria Antonini, Deborah Harvey, Emanuela Solano, An Van Breusegem, Arno Thomaes

**Affiliations:** 1 Research Institute for Nature and Forest (INBO), Geraardsbergen, Belgium; 2 Institute of Biological, Environmental and Rural Sciences (IBERS), Aberystwyth University, Penglais, Aberystwyth, United Kingdom; 3 Department of Biology and Biotechnology “Charles Darwin”, Sapienza - University of Rome, Rome, Italy; 4 School of Biological Sciences, Royal Holloway, University of London, Egham, Surrey, United Kingdom; 5 Research Institute for Nature and Forest (INBO), Brussels, Belgium; National Taiwan Normal University, TAIWAN

## Abstract

*Lucanus cervus* (L.), the stag beetle, is a saproxylic beetle species distributed widely across Europe. Throughout its distribution the species has exhibited pronounced declines and is widely considered threatened. Conservation efforts may be hindered by the lack of population genetic data and understanding of the spatial scale of population connectivity. To address this knowledge gap this research details the first broad scale phylogeographic study of *L*. *cervus* based on mitochondrial DNA (mtDNA) sequencing and microsatellite analysis of samples collected from 121 localities across Europe. Genetic data were complemented by palaeo-distribution models of spatial occupancy during the Last Glacial Maximum to strengthen inferences of refugial areas. A salient feature of the mtDNA was the identification of two lineages. Lineage I was widespread across Europe while lineage II was confined to Greece. Microsatellites supported the differentiation of the Greek samples and alongside palaeo-distribution models indicated this area was a glacial refuge. The genetic endemism of the Greek samples, and demographic results compatible with no signatures of spatial expansion likely reflects restricted dispersal into and out of the area. Lineage I exhibited a shallow star like phylogeny compatible with rapid population expansion across Europe. Demographic analysis indicated such expansions occurred after the Last Glacial Maximum. Nuclear diversity and hindcast species distribution models indicated a central Italian refuge for lineage I. Palaeo-distribution modelling results also suggested a western refuge in northern Iberia and south-west France. In conclusion the results provide evidence of glacial divergence in stag beetle while also suggesting high, at least on evolutionary timescales, gene flow across most of Europe. The data also provide a neutral genetic framework against which patterns of phenotypic variation may be assessed.

## Introduction

The stag beetle, *Lucanus cervus* L. (Coleoptera: Lucanidae) is distributed widely across Europe [1]. Past and current forest management practices, such as logging, wood harvesting, and removal of old trees and dead wood, have had detrimental effects on saproxylic biodiversity [2, 3]. In light of such declines the stag beetle is listed as “near threatened” in the European Red List [3] and has been protected by the Habitats Directive of the European Union since 1992 [4]. Conservation efforts may be hindered by a lack of information on the spatial scale at which populations may be connected on ecological and evolutionary timescales. Since dispersal distances in the species seem to range from a few hundred meters up to a maximum of five kilometres [5], distances between aggregates or populations cannot exceed these limits if sufficient gene flow is to be maintained within a metapopulation. Harvey et al. [1] reported that, since *L*. *cervus* shows an aggregated distribution, wood management may be critical for the species to counter local extinction. Furthermore, in species with presumed high levels of genetic structuring a significant loss of cryptic diversity is expected under changing conditions, such as climate change, when conservation policies do not incorporate these genetic differences [6, 7]. On a broader geographical scale the identification of intraspecific Evolutionarily Significant Units (ESUs), their evolutionary history and spatial distribution provide vital information to optimise conservation strategies which minimise genetic diversity loss at species level [8]. In addition to providing information for spatial management strategies, such phylogeographic approaches can also provide insight into how species have responded to historical climate change, and accordingly inform predictions of future climate change effects.

Pleistocene glaciations have influenced current patterns of genetic diversity and structure of fauna and flora [9, 10]. During the Last Glacial Maximum (LGM; 23 ky– 16 ky BP), substantial areas of northern Europe were covered by vast ice sheets, while permafrost reached most of continental Europe up to about 45°N [11]. During this period, southern European peninsulas (Iberian, Italian, Balkan) and the Caucasus/Caspian region acted as refugia where species persisted during glaciation, although additional glacial (cryptic) refugia and colonisation routes outside the Mediterranean region have been increasingly detected [12–15].

The sedentary nature of stag beetles [5, 16] is predicted to make them a model species for detecting signatures of historical climate driven processes [17]. Furthermore, as a result of their ecological specialization, it is probable that their colonisation process followed that of the broadleaf trees before and during the Pleistocene era [18]. The preferred habitat of the species is large diameter, decomposing logs which provide substantial substrate for habitat, have the capacity to hold moisture [19] and deliver more stable microclimatic conditions [20]. Moreover, such structures can persist for long periods within the landscape, potentially providing habitat for multiple generations which further ensures preservation of the phylogeographic signal in sedentary species.

To date, the genetic approach used in studies on *L*. *cervus* has been used to investigate processes like hybridisation [21] and localised intergenerational genetic patterns [22], or the congruence of morphological and molecular phylogenies at the (sub)species level [23]. The latter study has shown that certain subspecies, such as *L*. *c*. *akbesianus* from Turkey and *L*. *c*. *fabiani* (synonym *L*. *pontbrianti*) from France appeared to be different species, using cytochrome c oxidase subunit I (*COI*) sequences, while others could not be differentiated from *L*. *c*. *cervus* [23]. In this study we perform the first population genetic study of *L*. *c*. *cervus* at both regional and local geographical scales across Europe using both mtDNA and nuclear microsatellites. We combine genetic data with ecological niche modelling to robustly identify patterns of historical vicariance, glacial refugia locations and postglacial expansion dynamics. This study informs spatial conservation through identification of unique components of the species’ biodiversity and provides a valuable genetic baseline for future studies on the species.

## Material and methods

### Sampling and DNA-extraction

**Fig 1:**
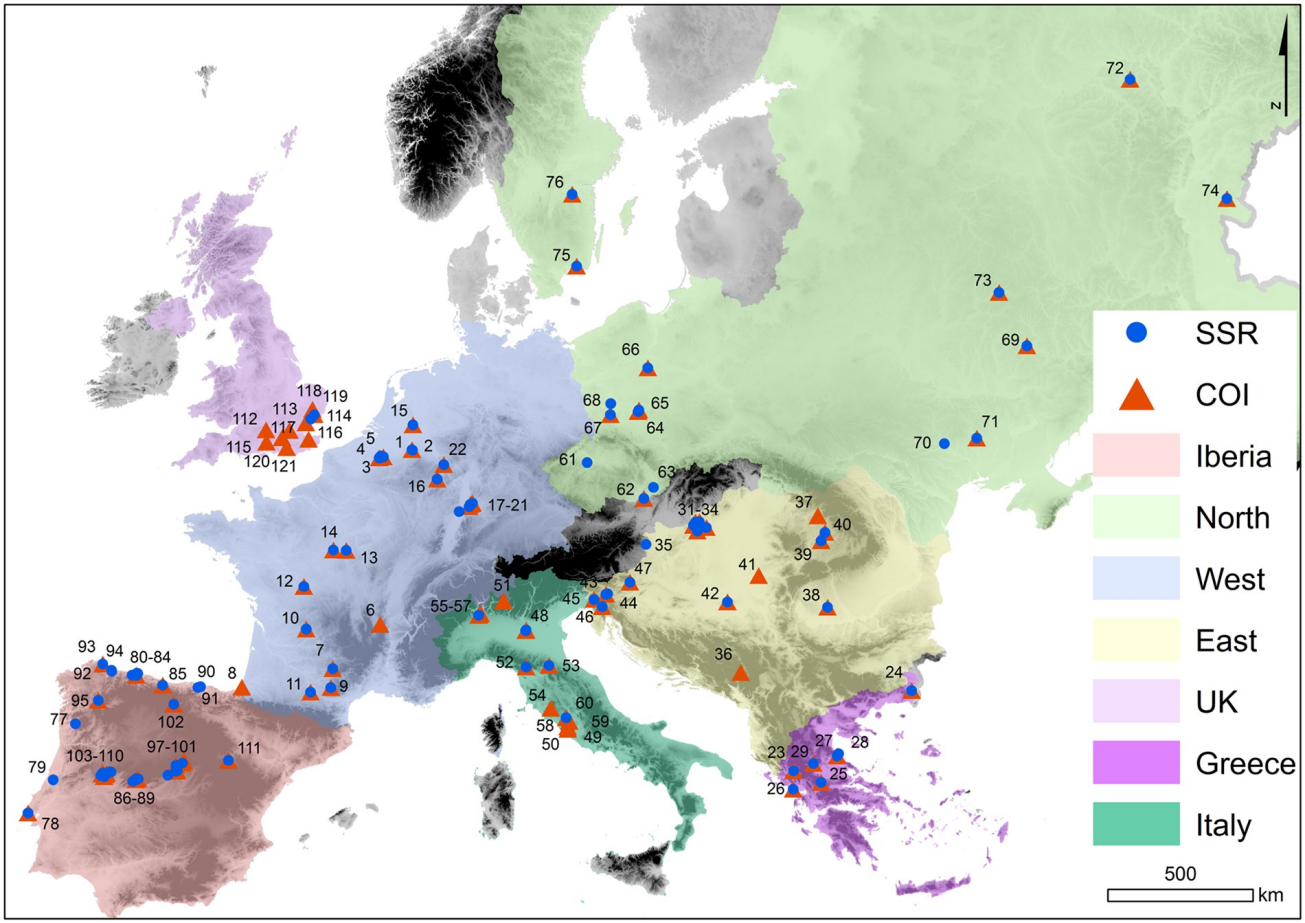
Sampling locations of *Lucanus cervus*. Localities with samples with *COI* sequences are indicated with orange triangles, those with samples genotyped with microsatellites with blue dots. The five regions over which the samples were divided are shown with different colours. The background is an altitude map of Europe in greyscale from the WorldClim database [26].

We requested samples from entomologists across Europe. Samples included whole beetles and parts of beetles, found as road kill or as predatory remains. In some cases a leg was removed from a live specimen, a treatment that does not seem to hinder further movement substantially [24, 25]. Many samples were part of an existing collection for which the entomologists required the necessary permits from national and/or local authorities when required. To ensure sampling of conspecifics and thus resolution of intraspecific processes sampling was restricted to *Lucanus cervus cervus*. Most samples were collected during the period 2001–2017, except for two samples from Craiova (Romania), collected in 1988, and six samples from Kursk (Russia), collected in 1990 (S1 Table). The samples originating from 121 localities (S1 Table, Fig 1) were either dried and kept at room temperature or preserved in absolute ethanol. The tissue used for DNA extraction depended on availability, but was mostly muscle tissue from legs. We extracted DNA from ground samples with the E.Z.N.A. Forensic DNA Kit (Omega Bio-Tek, VWR, Haasrode, Belgium) or the DNeasy Blood & Tissue Kit (Qiagen, Venlo, the Netherlands). The quality of DNA of 10% of the samples was assessed on 1% agarose gels. DNA quantification was performed with Quant-iT PicoGreen dsDNA Assay Kit (Life Technologies, Merelbeke, Belgium) using a Synergy HT plate reader (BioTek, BioSPX, Drogenbos, Belgium).

### Mitochondrial DNA

For the *COI* sequences, we followed the procedure reported by Cox et al. [23], including alignment and quality control. Several *COI* sequences available on GenBank were added (S1 Table): those obtained by Lin et al. [27], by Solano et al. [21] and by Cox et al. [23].

A haplotype network was constructed using the median-joining algorithm implemented in PopArt [28]. Phylogenetic analysis and the corresponding bootstrap analysis were performed using the maximum likelihood (ML) approach. For comparison, a Bayesian inference approach (BI) was also used. The ML analyses of unique haplotypes were achieved using RAxML 7.2.8 on RAxML BlackBox (http://phylobench.vital-it.ch/raxml-bb/index.php) [29] with 100 rapid bootstrap analyses followed by a search of the best-scoring ML tree in a single run. The general time reversible model (GTR) was used with an alpha parameter for the shape of the gamma distribution to account for among-site rate heterogeneity, as recommended by Stamatakis et al. [29].

The Bayesian analysis was conducted with a Markov chain Monte Carlo (MCMC) phylogenetic search using BEAST v. 2.4.8 [30]. We first identified the best fit model of molecular evolution under the Aikaike information criterion (AIC), as implemented in JModeltest v. 2.1.10 [31, 32]. Since the selected model, TIM2 + I + G (AIC = 4123.6848), was too complex to achieve convergence, the Hasegawa–Kishino–Yano (HKY) + G +I model was set instead. We applied a strict molecular clock with a rate of 1.77%/lineage per million years which was estimated for *COI* of tenebrionid beetles [33]. MCMC analyses were run for 5 x 10^8^ generations, sampling one tree every 1,000 generations. After verifying parameter convergence and effective sample size (>200) using Tracer v.1.6 [34], we discarded the first 10% of the trees as burn-in. A maximum clade credibility (MCC) tree was constructed using TreeAnnotator 2.4.8 [35] and visualised in FigTree v. 1.4.3 [36]. Owing to the computational limits using TreeAnnotator we first resampled one tree every 5 x 10^4^ generations using LogCombiner v. 2.4.8 [35].

The following diversity indices were calculated for each locality with at least five samples and for each geographic region using DnaSP v. 6.10.04 [37]: number of haplotypes (k), nucleotide diversity (π) and haplotype diversity (*h*). The seven geographic regions were defined to increase and balance sample sizes of the groups while taking the genetic structure (see below) into account (S1 Table, Fig 1). We call them ‘Greece’, ‘Italy’, ‘Iberia’, ‘UK’, ‘West’, ‘North’ and ‘East’ from now on. We assessed if there was a relationship between diversity indices and location (latitude and longitude) using Pearson correlation tests.

Population structure was investigated using the Bayesian program BAPS v. 6.0 [38]. We performed 10 runs for each K = 1–10. Since this resulted in an optimal cluster number of one, we wanted to assess additional or hierarchical genetic structure by fixing K = 2 and ran the analysis ten times to ensure convergence and consistency of the results. This was repeated for each inferred cluster. For populations with a minimum sample size of five individuals (with *COI* sequences), pairwise genetic differentiation among populations (F_ST_) was calculated using Arlequin v. 3.5.1.2 [39] with 10,000 permutations.

Several approaches were explored to investigate the demographic history of *Lucanus cervus*. First, we assessed population expansion of each cluster, as was defined by BAPS clustering results, and of the total dataset using Arlequin to calculate Tajima’s D [40] and Fu’s FS [41] with 2,000 simulated samples. If values are significantly different from zero, these neutrality tests indicate that populations deviate from expectations under a mutation–drift model. Next, a mismatch distribution analysis using Arlequin was performed for each lineage with 2,000 bootstrap replicates to evaluate the frequency distribution of the number of nucleotide mismatches among pairs of *COI* sequences. Whilst unimodal distributions represent expanding populations, populations of constant size show a multimodal distribution [42]. Additionally, we calculated the sum of square deviations (SSD) between the observed and the expected distribution and Harpending’s raggedness index (rg), which estimates the fluctuation in the frequency of pairwise differences [43].

To further assess past demographic dynamics through time, we ran a Bayesian skyline plot in BEAST with five group intervals for each lineage separately. All parameters were set identical to those described above (MCC tree), except for the number of MCMC in one of both lineages, which was 1 x 10^9^ for lineage I (see Results). Sampling of trees was done every 1 x 10^4^ generations. The median and corresponding credibility intervals of the Bayesian skyline plot were visualised with Tracer.

### Microsatellites

The DNA was eluted in 70 μl AE buffer. The integrity of DNA of 10% of the samples was assessed on 1% agarose gels. DNA quantification was performed with Quant-iT PicoGreen dsDNA Assay Kit (Life Technologies) using a Synergy HT plate reader (BioTek). Samples were genpotyped at the loci Lcerv-1, Lcerv-3, Lcerv-4, Lcerv-6, Lcerv-7, Lcerv-8, Lcerv-9, Lcerv-16, Lcerv-17, Lcerv-20, Lcerv-21, Lcerv-25, Lcerv-28, Lcerv-29, Lcerv-30, Lcerv-31 and Lcerv-36, described by McKeown et al. [44]. The primer sets were included in four multiplex PCRs and one simplex PCR (S2 Table). The multiplex PCR contained 1 μl DNA solution (5–10 ng/μl), 5 μl Multiplex PCR Master Mix (Qiagen) and a certain concentration of each primer pair shown in S2 Table. Autoclaved ultrapure water was added to a total volume of 10 μl. PCR conditions were as follows: 15’ at 94°C, 35 cycles of 30” at 94°C, 30” at annealing temperature (S2 Table) and 30” at 72°C. The final extension step was carried out during 10’ at 72°C, ending with 15’ at 4°C, after which temperature was kept at 15°C. We performed the genotyping analysis on an ABI 3500 Genetic Analyzer (Applied Biosystems) with the GeneMapper v.4.0 software package. To test for reproducibility, 6% of the samples were blindly replicated two to six times within and across well plates. Samples with fewer than 12 scored loci were discarded. To investigate possible deviations from Hardy–Weinberg equilibrium (HWE), we used the test available in the program Genepop 4.6 [45]. To take in account population structure, we reduced the dataset to those localities with five samples or more. Genepop was also used to assess the presence of null alleles with the maximum likelihood method following the expectation maximization (EM) algorithm of Dempster et al. (1977), and to test for linkage disequilibrium (LD) for each pair of loci. We implemented a correction for multiple testing with the false discovery rate method (FDR) [46] with a nominal level of 5%. To assess the influence of loci with potential null alleles on population structure analyses, we calculated pairwise F_ST_ values with and without such loci using Genalex v. 6.503 [47, 48].

As with the mitochondrial sequences, we applied the BAPS v. 6.0 spatial clustering of individuals [49]. The program was run ten times for each value of K = 1–39. An admixture analysis [50] was performed using 100 iterations, a minimum of three individuals per population, 200 reference individuals for each population, and 20 iterations of reference individuals. We used another Bayesian approach implemented in STRUCTURE v. 2.3.4 [35] with K 1 to 20, replicating each value of K three times. The simulations were run with a burn-in and run length each of 1 x 10^5^ iterations. We chose the admixture model and used the correlated allele frequency option and the localities as prior information (locprior). The optimal K was determined based on the average natural logarithm of the probability of the data (Ln Pr(X|K)) and the Evanno method [51] in STRUCTURE HARVESTER v. 0.6.94 [52]. As the ΔK method of Evanno et al. [51] is likely to identify the uppermost hierarchical level of population structure, subsequent hierarchical analysis was performed. Result files from STRUCTURE HARVESTER were processed in CLUMPP v. 1.1.2 [53]. Furthermore, pairwise F_ST_ values were calculated among populations with at least five samples and visualised in a principal coordinate analysis (PCoA) using Genalex v. 6.503. Significance of these values was assessed using 9,999 permutations.

We calculated the following estimates of genetic diversity using the R package ‘*hierfstat*’ v. 0.04–22 [54] for the localities with at least five genotyped individuals: rarefied allelic richness (A_R_), observed (H_O_) and expected heterozygosity (H_E_). Also the fixation index F_IS_ was estimated. The same measures were estimated for the different predefined geographical regions (Fig 1) following partly the structure results as well as to obtain more balanced sample sizes. We evaluated if a relationship exists between latitude/longitude and the level of diversity as measured with H_E_ and A_R_ through Pearson correlation tests. Additionally, we tested if isolation-by-distance (IBD) was present with a Mantel test based on 9,999 replicates implemented the R package ‘*adegenet*’ v. 2.0.1 [55].

### Palaeo-distribution modelling

Modelling of the geographical distribution of *L*. *cervus* was performed using MaxEnt v. 3.4.1 [56] implemented in the R package ‘*dismo*’ v. 1.1–4 [57], based on current conditions and then projected onto two palaeoclimatic models for the LGM (~22 ky BP), the Community Climate System Model (CCSM4) and the Model for Interdisciplinary Research On Climate (MIROC-ESM).

Species records from the Global Biodiversity Information Facility (GBIF) [58] complemented our own records (including non-genotyped samples). We filtered the GBIF records to those having unique coordinates with a precision of less than 2 km. We first randomly selected one record in each grid cell with size 2.5 arc minutes (approximately 5 km) using the R package ‘*GSIF*’ v. 0.5–4 [59], which corresponds with the resolution of the climatic data we downloaded from the WorldClim database [26]. Since GBIF records were highly concentrated in Germany, England and Sweden we further thinned the occurrences using the R package *‘spThin’* v. 0.1.0 [60] which returns the largest number of records that are no closer to each other than a user-defined linear distance. We chose a minimum nearest neighbour distance of 5 km and repeated the analysis 200 times to obtain the highest number of occurrences possible. Since we had a high number of records, we applied the thinning procedure to four regions separately: England, Sweden, Germany and the remaining area. We used 19 bioclimatic variables available at WorldClim and the complementary ENVIREM dataset that comprises 16 climatic and two topographic variables [61]. Each layer was cropped to an extent (-15° to 55° E, 30° to 65° N) that is slightly larger than that of the current distributional range (-13° to 43° E, 35° to 62° N). A set of 10,000 random pseudo-absence records was created within this extent. We built the MaxEnt model using the R package ‘*MaxentVariableSelection*’ v. 1.0–3 [62] that reduces the set of variables in a stepwise manner to avoid overfitting the model to the occurrence data. Variables that contributed less than 3% to the model were removed, while also keeping the Pearson correlation values below 0.9. After each step, model performance is assessed based on the sample size adjusted Akaike information criterion (AICc), based on all occurrence locations, and the area under the receiver operating characteristic (AUC) based on 50% test data and 10 replicate runs. We selected the model with the lowest AICc value to obtain a model that identifies the fundamental niche of stag beetle and that is better transferable to other climate scenarios [63, 64]. A range of regularisation multiplier (β) values, from 1 to 15 in increments of 0.5, were simultaneously tested.

In order to assess if the analysis region during the LGM has similar environmental conditions as it has at present and if extrapolation risks exist, we used the mobility-oriented parity (MOP) metric [65] implemented in the R package *‘kuenm’* v. 1.1.1 (https://github.com/marlonecobos/kuenm) [66].

## Results

### Mitochondrial DNA

**Fig 2:**
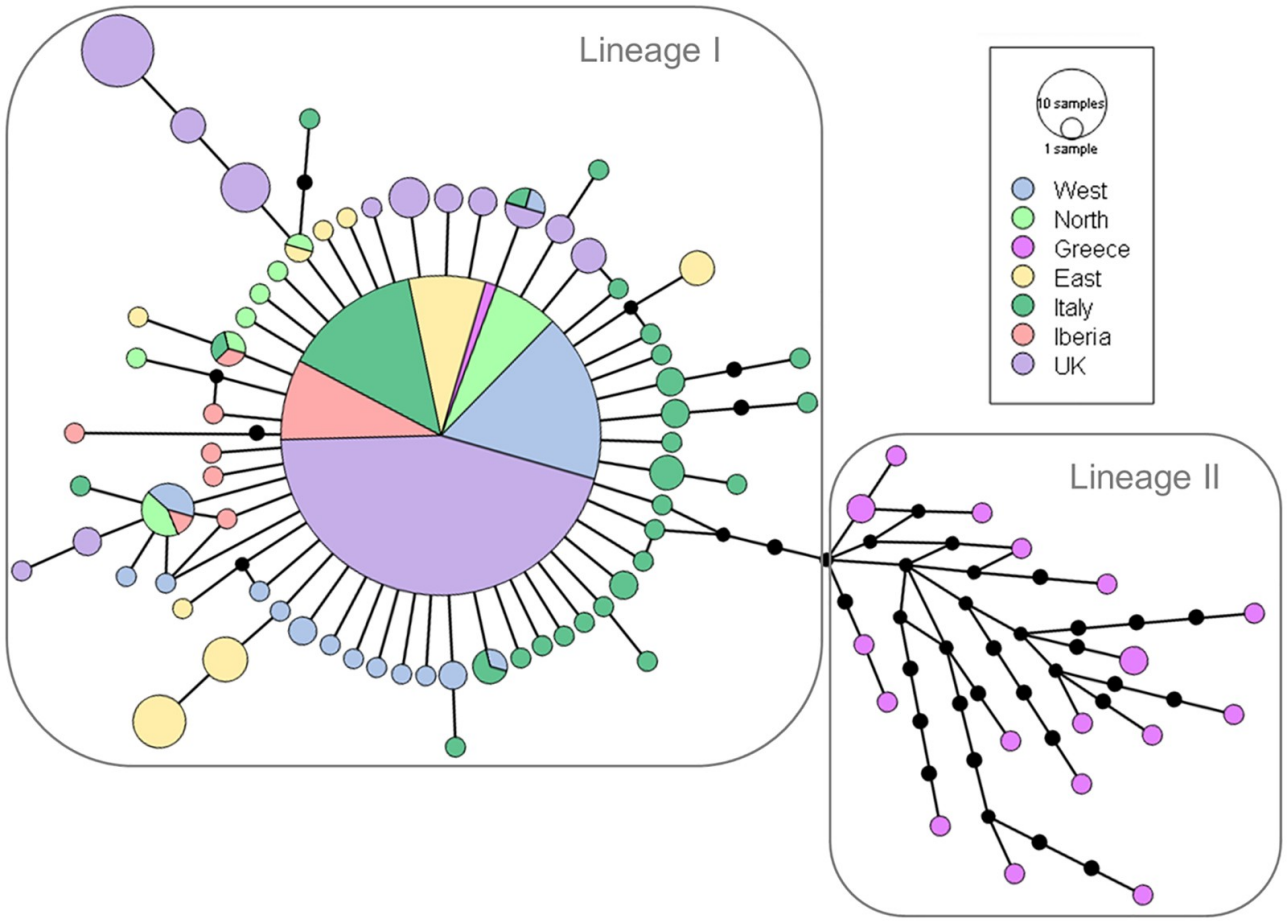
Median joining haplotype network of mitochondrial cytochrome oxidase subunit I (*COI*) haplotypes of *Lucanus cervus* in Europe. Partitions inside the circles represent the proportion of each region as given in Fig 1 within each haplotype. Circle size is proportionate with sample size. Haplotypes connected by a line differ in sequence by one mutational step and black dots represent unsampled or extinct haplotypes.

**Fig 3:**
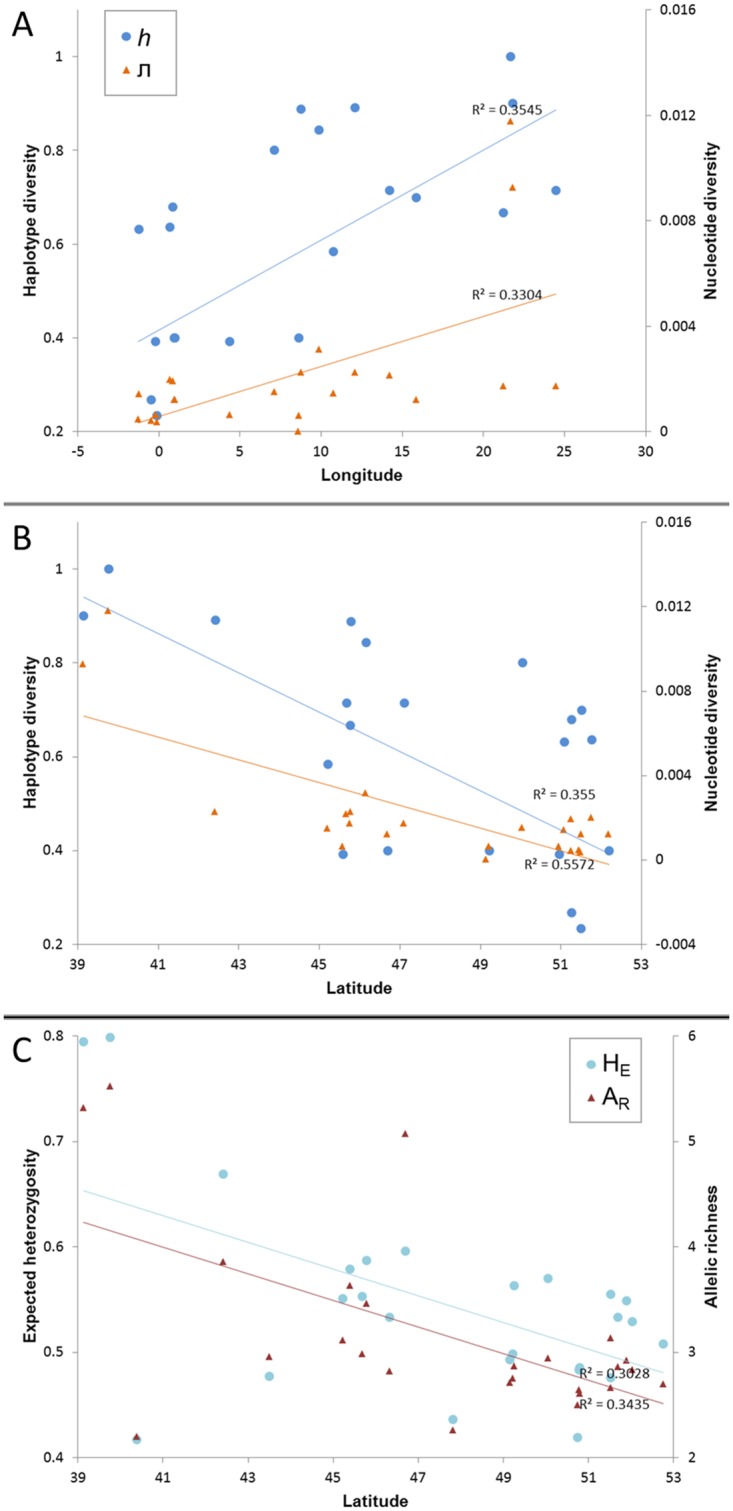
Population-level genetic diversity in relation to latitude and longitude. (A) Haplotype diversity (*h*) and nucleotide diversity (ᴫ) based on *COI* sequences in relation to longitude and (B) in relation to latitude. (C) Expected heterozygosity (H_E_) and allelic richness (A_R_) based on microsatellite genotypes in relation to latitude.

In total, we obtained 404 sequences of specimens from localities spread across the entire distribution range of *L*. *cervus* (Fig 1). Sequences were submitted to GenBank (S1 Table). Only 85 haplotypes were detected, defined by 83 polymorphic sites of which 43 parsimony informative. The haplotype network conformed to a star-like topology consisting of a central and most abundant haplotype from which many low frequency haplotypes radiated, separated by usually one to two substitutions (lineage I, Fig 2). The central haplotype was found across Europe. Some Italian and samples of region ‘East’ differed by three mutational steps from the main haplotype, while some ‘UK’ samples differed by three or four steps. A Greek lineage (lineage II) is separated by five mutations from the dominant haplotype which harbours a wide variety of rather distantly related haplotypes. Only three samples of ‘Greece’ shared the main European haplotype. In general, diversity was relatively high, particularly in southern and eastern regions (Table 1), with the highest values for number of haplotypes (k), π and *h* in ‘Greece’. In ‘Italy’ π and *h* were lower and comparable to the levels of ‘East’. The lowest levels in diversity seemed to be in regions ‘West’, ‘UK’ and ‘Iberia’. These patterns were evident in correlation tests with a positive relationship between π or *h* and longitude (r = 0.57, p = 0.004 and r = 0.60, p = 0.003, respectively), while diversity decreased with increasing latitude (r = -0.75, p = 4.3 x 10^−5^ and r = -0.60, p = 0.003, for π and *h* respectively) (Fig 3A and 3B).

**Table 1:** Genetic diversity indices for regions and localities with a minimum sample size of five individuals at 16 microsatellite loci and/or based on *COI* sequences.

Region	Country	Locality	N_coi	N_ssr	k	π	*h*	H_O_	H_E_	A_R_	F_IS_
East			40	34	10	0.00282 (0.00035)	0.737 (0.061)	0.509 (0.225)	0.555 (0.171)	4.374 (1.626)	0.073 (0.243)
	Romania	Bistrita	7	0	3	0.00171 (0.00059)	0.714 (0.127)				
		Târnăveni	2	5				0.538 (0.299)	0.533 (0.195)	2.812 (0.834)	-0.004 (0.371)
		Timisoara	7	0	3	0.00171 (0.00066)	0.667 (0.160)				
	Slovenia	Pivka	7	9	3	0.00213 (0.00043)	0.714 (0.127)	0.549 (0.274)	0.553 (0.189)	2.976 (1.035)	0.005 (0.355)
Greece			22	28	18	0.01090 (0.00073)	0.978 (0.021)	0.723 (0.177)	0.811 (0.131)	9.954 (4.704)	0.093 (0.226)
	Greece	Neraida	5	5	4	0.00925 (0.00205)	0.900 (0.161)	0.750 (0.258)	0.795 (0.164)	5.312 (1.778)	0.023 (0.343)
		Vlahava	8	12	8	0.01178 (0.00157)	1 (0.063)	0.719 (0.187)	0.799 (0.161)	5.514 (1.518)	0.079 (0.223)
Iberia			28	58	9	0.00096 (0.00028)	0.497 (0.117)	0.501 (0.244)	0.508 (0.179)	4.075 (1.477)	0.030 (0.196)
	Spain	Dehesa de Robleda	3	5				0.453 (0.390)	0.417 (0.272)	2.188 (1.047)	-0.093 (0.565)
		La Laguna	0	6				0.490 (0.240)	0.477 (0.218)	2.946 (1.279)	-0.042 (0.178)
Italy			69	57	31	0.00216 (0.00030)	0.745 (0.059)	0.543 (0.239)	0.597 (0.162)	5.259 (1.815)	0.097 (0.270)
	Italy	Acquapendente	11	8	8	0.00224 (0.00054)	0.891 (0.008)	0.574 (0.252)	0.669 (0.117)	3.850 (1.040)	0.152 (0.308)
		Bernate	10	9	6	0.00224 (0.00050)	0.889 (0.091)	0.576 (0.279)	0.587 (0.198)	3.454 (1.237)	0.018 (0.293)
		Marmirolo	20	34	7	0.00145 (0.00046)	0.584 (0.127)	0.540 (0.258)	0.551 (0.170)	3.108 (0.878)	0.019 (0.328)
		Sondrio	10	0	6	0.00312 (0.00082)	0.844 (0.103)				
North			26	76	9	0.00135 (0.00034)	0.622 (0.107)	0.486 (0.265)	0.509 (0.194)	3.750 (1.356)	0.079 (0.344)
	Poland	Janikow	2	5				0.462 (0.356)	0.508 (0.238)	2.687 (1.014)	0.111 (0.530)
		Milicz	3	5				0.512 (0.342)	0.555 (0.268)	3.125 (1.500)	0.059 (0.419)
		Pnewkow	5	37	3	0.00119 (0.00045)	0.700 (0.218)	0.506 (0.282)	0.476 (0.215)	2.653 (0.905)	-0.062 (0.323)
	Russia	Kursk	3	6				0.490 (0.336)	0.533 (0.226)	2.856 (1.003)	0.048 (0.494)
	Ukraine	Berezovka	4	5				0.400 (0.358)	0.436 (0.210)	2.250 (0.683)	0.077 (0.628)
West			62	144	15	0.00095 (0.00019)	0.497 (0.079)	0.482 (0.228)	0.539 (0.133)	4.091 (1.412)	0.110 (0.271)
	Belgium	Overijse	2	30				0.471 (0.256)	0.483 (0.144)	2.633 (0.737)	0.061 (0.294)
		Sint-Genesius-Rode	2	10				0.447 (0.296)	0.419 (0.205)	2.492 (0.840)	-0.031 (0.302)
		Watermaal-Bosvoorde	0	30				0.463 (0.230)	0.485 (0.177)	2.603 (0.861)	0.032 (0.275)
	France	Bussiere	18	0	3	0.00062 (0.00023)	0.392 (0.018)				
		Lanouaille	3	5				0.606 (0.259)	0.579 (0.128)	3.625 (3.117)	-0.044 (0.408)
		Lurais	5	5	2	0.00119 (0.00071)	0.400 (0.237)	0.550 (0.306)	0.596 (0.144)	5.063 (8.575)	0.068 (0.517)
	Germany	Alf	6	5	4	0.00149 (0.00045)	0.800 (0.172)	0.513 (0.318)	0.570 (0.169)	2.938 (0.854)	0.082 (0.470)
		Forst	5	10	1	0	0	0.469 (0.309)	0.493 (0.212)	2.700 (1.024)	0.044 (0.420)
		Kronau	5	15	2	0.00060 (0.00035)	0.400 (0.237)	0.503 (0.306)	0.498 (0.199)	2.741 (0.904)	-0.005 (0.386)
		Tairnbach	2	13				0.500 (0.250)	0.563 (0.125)	2.861 (0.800)	0.093 (0.391)
UK			157	19	15	(0.00129 0.00016)	0.474 (0.049)	0.515 (0.276)	0.554 (0.147)	3.904 (1.689)	0.101 (0.338)
	United Kingdom	Berkshire	20	0	2	0.00045 (0.00039)	0.100 (0.088)				
		Colchester	3	13				0.522 (0.283)	0.549 (0.165)	2.918 (0.887)	0.064 (0.338)
		Copdock	4	6				0.500 (0.333)	0.529 (0.177)	2.823 (0.871)	0.088 (0.493)
		Essex	20	0	3	0.00197 (0.00020)	0.637 (0.064)				
		Hampshire	20	0	5	0.00141 (0.00036)	0.632 (0.112)				
		Kent	20	0	5	0.00190 (0.00029)	0.679 (0.080)				
		London	16	0	2	0.00035 (0.00019)	0.233 (0.126)				
		Suffolk	16	0	2	0.00119 (0.00034)	0.400 (0.114)				
		Surrey	20	0	2	0.00040 (0.00017)	0.268 (0.113)				
		Sussex	18	0	3	0.00062 (0.00023)	0.392 (0.133)				

N_coi, number of samples with sequences of *COI*; N_ssr, number of samples with microsatellite genotypes; k, number of haplotypes; π, nucleotide diversity; *h*, haplotype (gene) diversity; H_O_, observed heterozygosity; H_E_, expected heterozygosity; A_R_, allelic richness rarefied at 10 for localities and at 36 for regions; F_IS_, fixation index; standard deviations between brackets.

**Fig 4:**
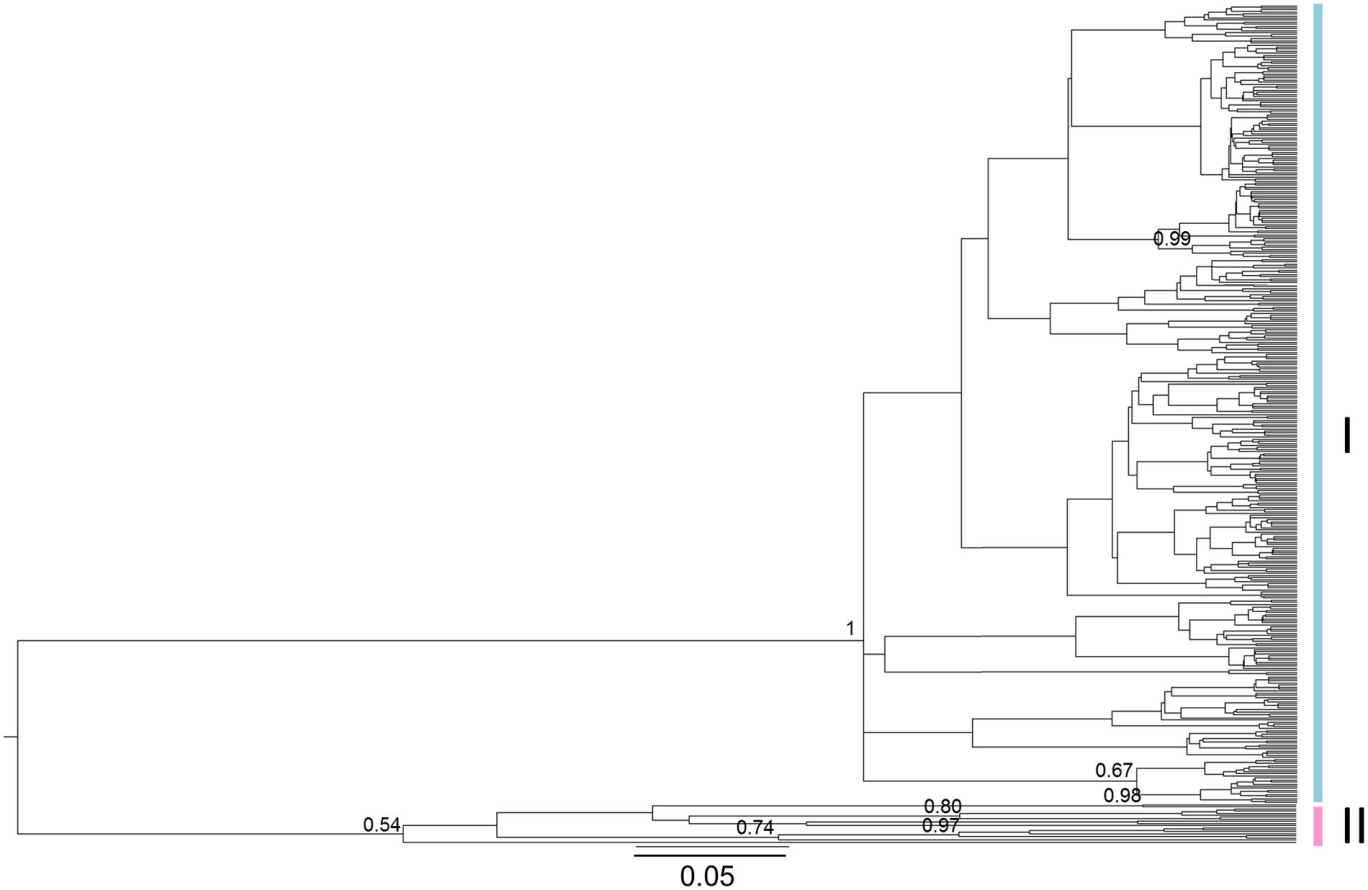
Bayesian maximum clade credibility tree of the mtDNA dataset of *Lucanus cervus* obtained using BEAST. Bayesian posterior probabilities higher than 0.5 are shown on nodes. Lineage I is indicated in blue and lineage II in pink.

**Fig 5:**
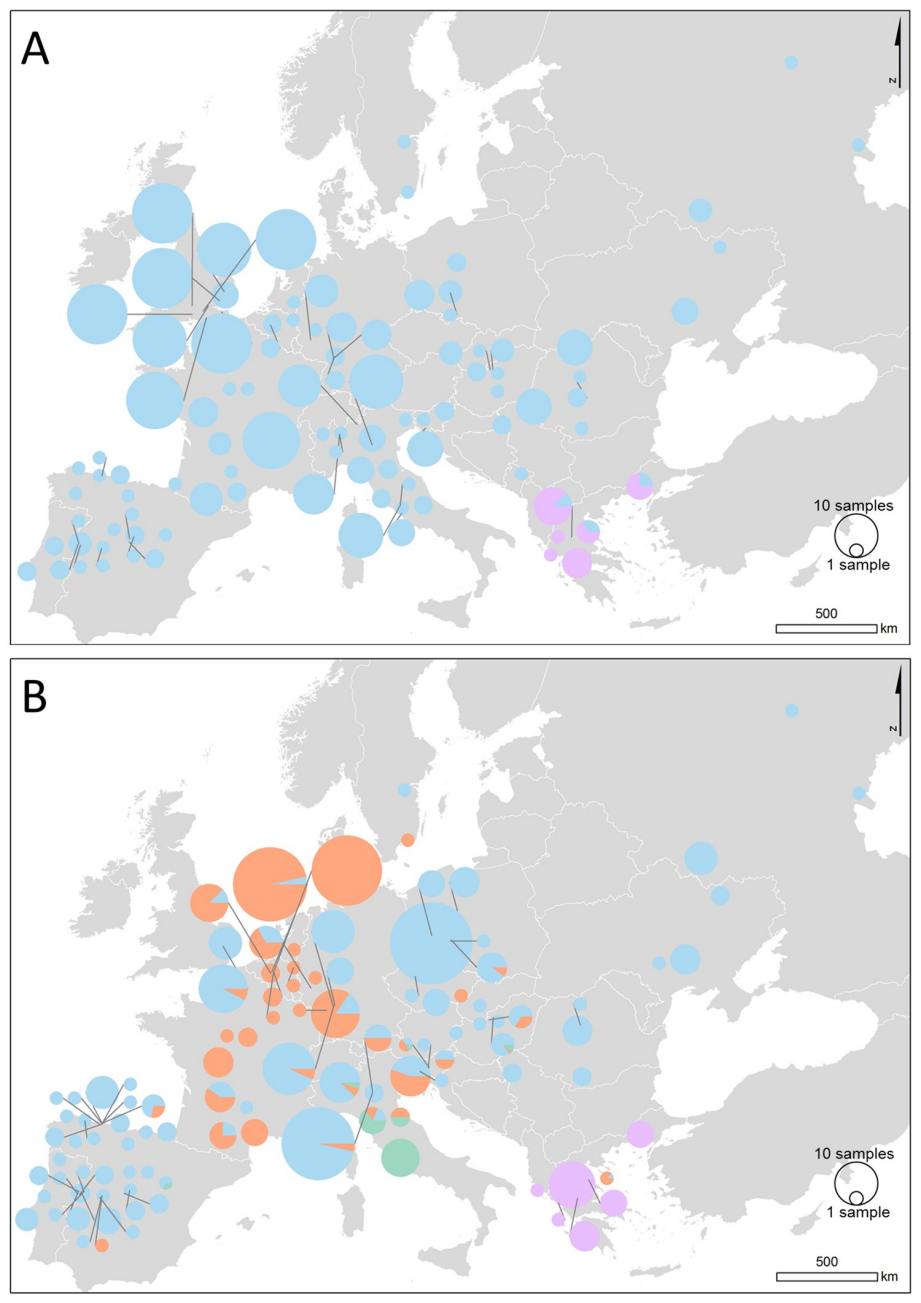
Bayesian cluster memberships obtained using BAPS for (A) the mtDNA dataset of *Lucanus cervus* and (B) the microsatellite genotypes. The pie charts represent the distribution of BAPS clusters, given in different colours, and are scaled according to sample size.

According to the ML tree the haplotypes spread across Europe formed a clade with moderate support and was separated from the majority of the Greek haplotypes. The latter, however, seemed to be paraphyletic (S1 Fig). This changed using the Bayesian approach with all samples included. In the MCC tree, the Greek haplotypes (except for three samples with the main haplotype) formed a monophyletic clade but with low support (0.54), while the European haplogroup had high support (1; Fig 4). No apparent geographical structure was present within both clades. As expected, the Bayesian clustering analysis with K fixed as 2 resulted in the same two groups: a cluster in Greece and a second with individuals all over Europe including some in Greece (Fig 5A). No further hierarchical clustering was found.

High F_ST_ values (0.380–0.831) were found among populations of Greece (Neraida and Vlahava) and the remaining populations, representing the two lineages (S3 Table). Populations of region ‘East’ seemed also well differentiated, especially Bistrita and Timisoara in Romania (F_ST_ = 0.488–0.787). Other population comparisons resulted in lower to non-significant F_ST_ values, except for the population from Essex which was well differentiated, even from other populations in the ‘UK’ region (F_ST_ > 0.193), Kent not included (F_ST_ = 0.039).

**Fig 6:**
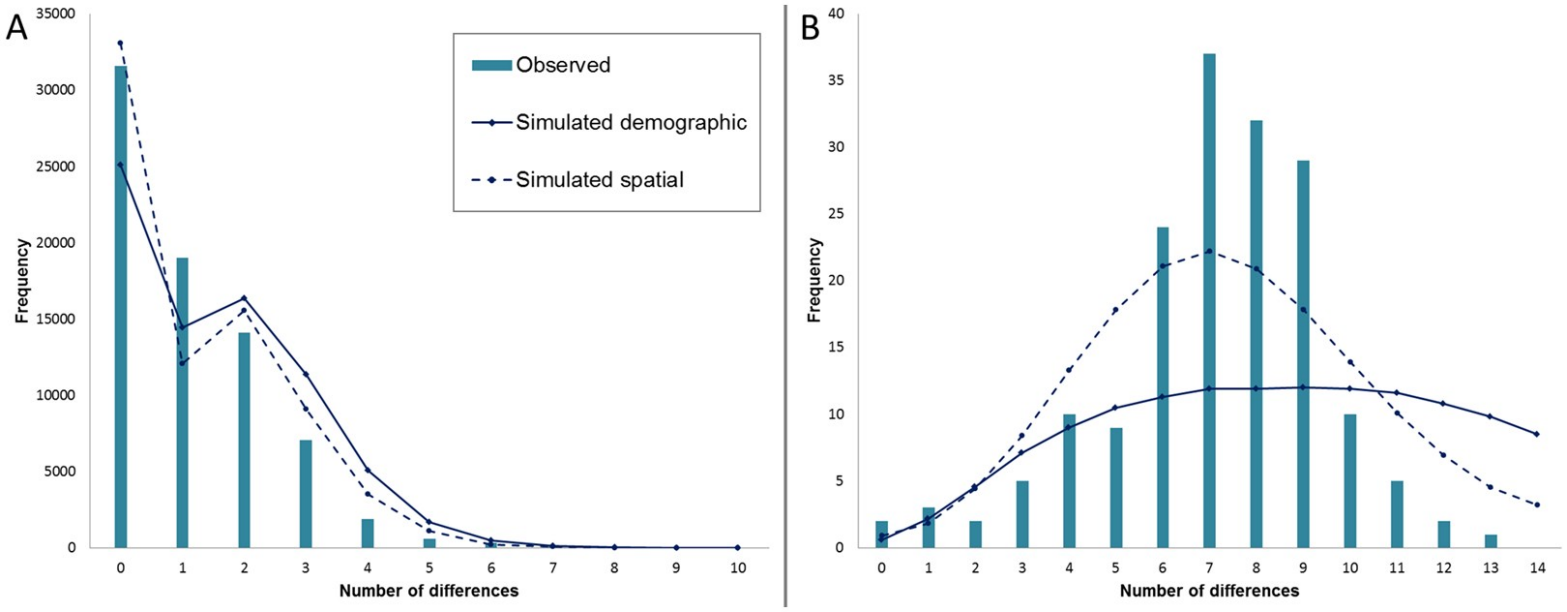
Mismatch distribution of *Lucanus cervus* mtDNA in lineage I (A) and lineage II (B). The x-axes display the number of nucleotide differences.

**Fig 7:**
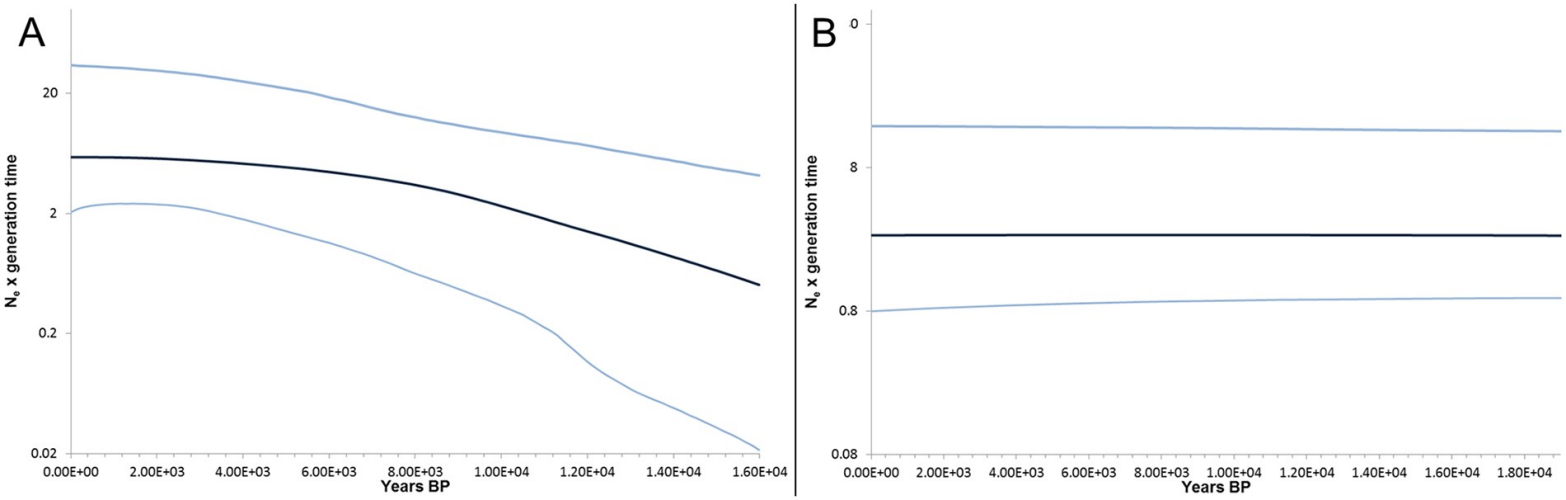
Bayesian skyline plot of *Lucanus cervus* created with the *COI* dataset. The y-axis represents the product of effective population size (N_e_) and generation time (with units in millions of years) on a logarithmic scale. The black line gives the median of effective population size through time and the blue lines represent the 95% highest posterior densities over the median estimates along the coalescent history. The x-axis gives years before present.

In lineage I, Tajima’s D and Fu’s FS were significantly negative indicating demographic expansion, while only the latter was significant in lineage II (Table 2). Furthermore, the SSD under the demographic expansion model was significant in lineage II which suggests population stability as is shown in the mismatch distribution in Fig 6B. The significant value of SSD under the spatial expansion model in Greece further indicates no range expansion took present. The mismatch distribution of lineage I followed an L-shaped, positively skewed distribution which is expected for star-like topologies (Fig 6A). Demographic and/or spatial expansion in this cluster seemed to have occurred. The BSP also supports a population expansion in lineage I (Fig 7A) and a stable population size through time in lineage II (Fig 7B). The start of the population expansion of lineage I occurred around 16 ky before present, after the LGM.

**Table 2:** Results of demographic analyses on *COI* sequences in the two lineages of *Lucanus cervus*.

	D	FS	SSD_demo	SSD_spat	rg
Lineage I	-2.628***	-28.390***	0.0012	0.0001	0.0468
Lineage II	-1.153	-8.604**	0.0424***	0.0166*	0.0295
Total	-2.537***	-26.918***	0.0024	0.0021	0.0387

D, Tajima’s D; FS, Fu’s FS; SSD_demo, sum of squared deviation under the sudden, demographic expansion model; SSD_spat, sum of squared deviation under the spatial expansion model; rg, Harpending’s raggedness index.

* significant at P < 0.05

** significant at P < 0.0

*** significant at P < 0.001

### Microsatellites

Eight loci deviated from HWE in one to three localities of which four in Marmirolo and three in Vlahava. The locus deviating from HWE in three localities (Watermaal-Bosvoorde, Aquapendente and Vlahava), Lcerv-30, also showed an elevated proportion of null alleles (> 20%) in 13 localities, while loci Lcerv-16 and Lcerv-29 showed such levels in respectively seven and eight localities. There was no sign of LD among pairs of loci. We discarded locus lcerv-30, but tested for changes in population structure through the calculation of pairwise F_ST_ values after excluding Lcerv-16, Lcerv-29 or both. Because F_ST_ values hardly changed, both were included for further analysis. Mean error rate was 4% and no samples were discarded of a total of 416.

#### Genetic structure and diversity

The spatial clustering analysis using BAPS resulted in an optimum of four clusters (Fig 5B). Some admixture was found in only a limited number of samples, of which the majority (4 samples) was located in northern Italy (S2A Fig). Except for one, individuals from Greece formed a separate group (i.e. ‘cluster 1’). The second cluster consisted of individuals from Central Italy and some from northern Italy (‘cluster 2’). Low levels of admixture (between 19% and 33%) with this Italian gene pool also occurred in Slovenia, Hungary and Spain. Another spatial group (‘cluster 3’) predominated in the regions ‘East’, ‘North’, ‘Iberia’, ‘UK’, the north of ‘Italy’ and, to some extent, in ‘West’, especially in Germany (Forst, Rettigheim and Kronau). Finally, the fourth cluster was mainly located in region ‘West’ with some individuals scattered in the other regions (‘cluster 4’). Some localities showed a mixture of individuals assigned to this fourth and the widespread cluster 3. The same four clusters were suggested by BAPS after randomly reducing the sample sizes in two Belgian localities from 30 to 10 individuals. It therefore does not seem that the larger sample sizes at these localities could have influenced results. Initially, STRUCTURE analysis resulted in an optimal K = 2 (S3A Fig). The same Greek cluster was formed as with BAPS, while the remaining samples were mostly assigned to a second cluster (S2B Fig). Subsequent analysis for substructure in this second cluster delivered again the highest ΔK for K = 2 subclusters (S3B Fig). A second peak in ΔK was obtained at K = 4 subclusters (S3B Fig), where also the average Ln Pr(X|K) values started to plateau. Results for both K values are included in S2(C) and S2(D) Fig. For K = 2 subclusters, samples were divided in the two clusters 3 and 4 as defined by BAPS, but with the Italian samples assigned to cluster 3. For K = 4 subclusters, the samples from Central Italy form a separate subcluster, very similar to cluster 2 according to the BAPS results (S2D Fig). A fourth subcluster consisted substantially out of admixed individuals, with likelihood admixture ratios > 0.5, particularly in two localities Watermaal-Bosvoorde (Belgium) and Tairnbach (Germany) with a mean ratio of 0.566 and a range of 0.251–0.724. We did not investigate further subclustering because of the low sample sizes in many localities.

**Fig 8:**
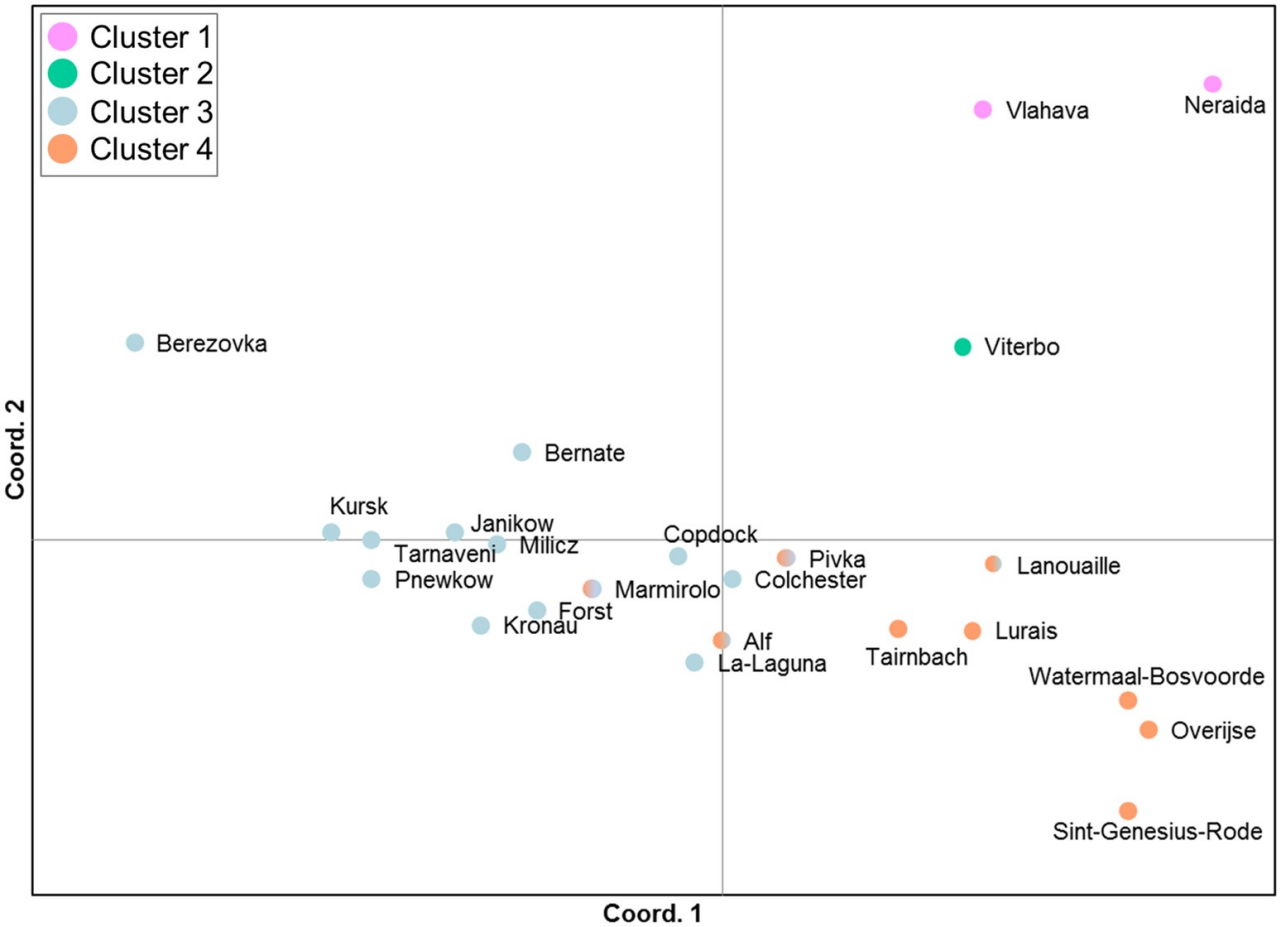
Principal Coordinates Analysis (PCoA) of population pairwise F_ST_ values for microsatellite genotypes of *Lucanus cervus*. The dots are coloured according to the clusters defined by BAPS (see Fig 5B and S2 Fig).

Pairwise F_ST_ values ranged from 0.025 to 0.207 (S4 Table). The pattern in F_ST_ values followed largely the Bayesian clustering results (Fig 8), differentiating the Greek (pairwise F_ST_ values for Vlahava: 0.093–0.176; for Neraida: 0.116–0.202) and central Italian populations (pairwise F_ST_ values for Viterbo: 0.086–0.174). However, the Berezovka population in Ukraine from region ‘North’ displayed high F_ST_ values, although it was assigned to the most abundant and wide spread cluster. Sample size for this population was, however, only five.

Gene diversity and allelic richness reached the highest values in ‘Greece’ and the second highest in ‘Italy’ (Table 1). In the other regions levels of genetic variation were similar, although values for both H_E_ and A_R_ were quite high in Lanouaille and Lurais in Central France of region ‘West’ (H_E_ ranged from 0.579 to 0.596 and A_R_ from 3.625 to 5.063). Multilocus F_IS_ values were not significant in any sample. The H_E_ and A_R_ decreased with increasing latitude (r = -0.55, p = 0.004 and r = -0.59, p = 0.002, respectively) (Fig 3C), but no significant pattern with longitude was detected (r = 0.27, p = 0.198 and r = 0.12, p = 0.561, respectively). The relationship between genetic and geographic distance was weak but significant (r = 0.18, p = 0.0001). However, IBD was not significant when Greek samples were excluded (r = 0.036, p = 0.075).

### Palaeo-distribution modelling

A total of 37,851 occurrence records was reduced to 16,493 records having unique coordinates with a precision of at least 2 km. After filtering these records to one occurrence per grid cell of 2.5 arc minutes, a total of 2325 points remained. This number dropped to 1279 observations when thinned using a nearest neighbour distance of 5 km (S4 Fig). The model with the lowest AICc had a regularisation multiplier of 1 and only four environmental variables: precipitation of the driest month, Thornthwaite aridity index (index of the degree of water deficit below water need), continentality (average temperature of warmest month—average temperature of coldest month) and maximum temperature of the coldest month. The average AUC test value for this model was 0.927 that differed on average 0.003 from the AUC training value. The most important variable was continentality with a model contribution of 43%, while the second most important was precipitation of the driest month which contributed 33% to the model. The variables ‘Thornthwaite aridity index’ and ‘maximum temperature of the coldest month’ had a relative contribution score of 14% and 10%, respectively.

**Fig 9:**
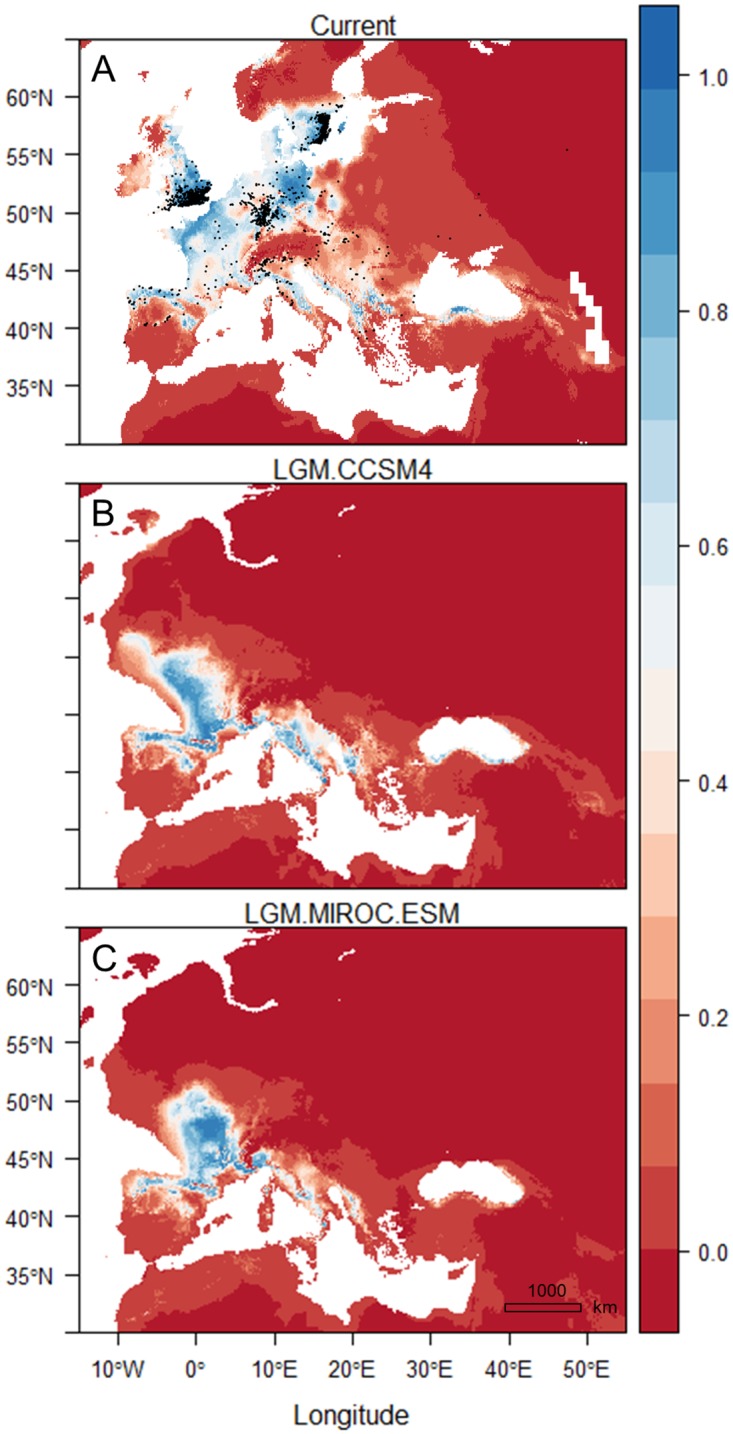
Species distribution models using 1279 occurrence data points of *Lucanus cervus*. (A) The current distribution with the occurrence records (black dots), the distribution during the Last Glacial Maximum based on two general circulation models, (B) CCSM4 and (C) MIROC-ESM. The legend indicates low (0) to high (1) environmental suitability for *L*. *cervus*.

The present species distribution model encompassed mainly the north-western part of Europe, but also northern Iberia, parts of Italy, east of the Adriatic Sea through to Albania and the north of Greece, Serbia and Bulgaria, and the Black Sea coast of Turkey (Fig 9A). When projected to the conditions of the CCSM4 scenario of LGM, suitable habitat seemed to be restricted to the southern parts of current distribution, with an increase in southern France and in Italy (Fig 9B). A decrease in habitat appeared in the north-eastern and eastern Mediterranean. The MIROC-ESM scenario showed the same trend but with smaller areas of suitable habitat in aforementioned regions and in Italy (Fig 9C).

**Fig 10:**
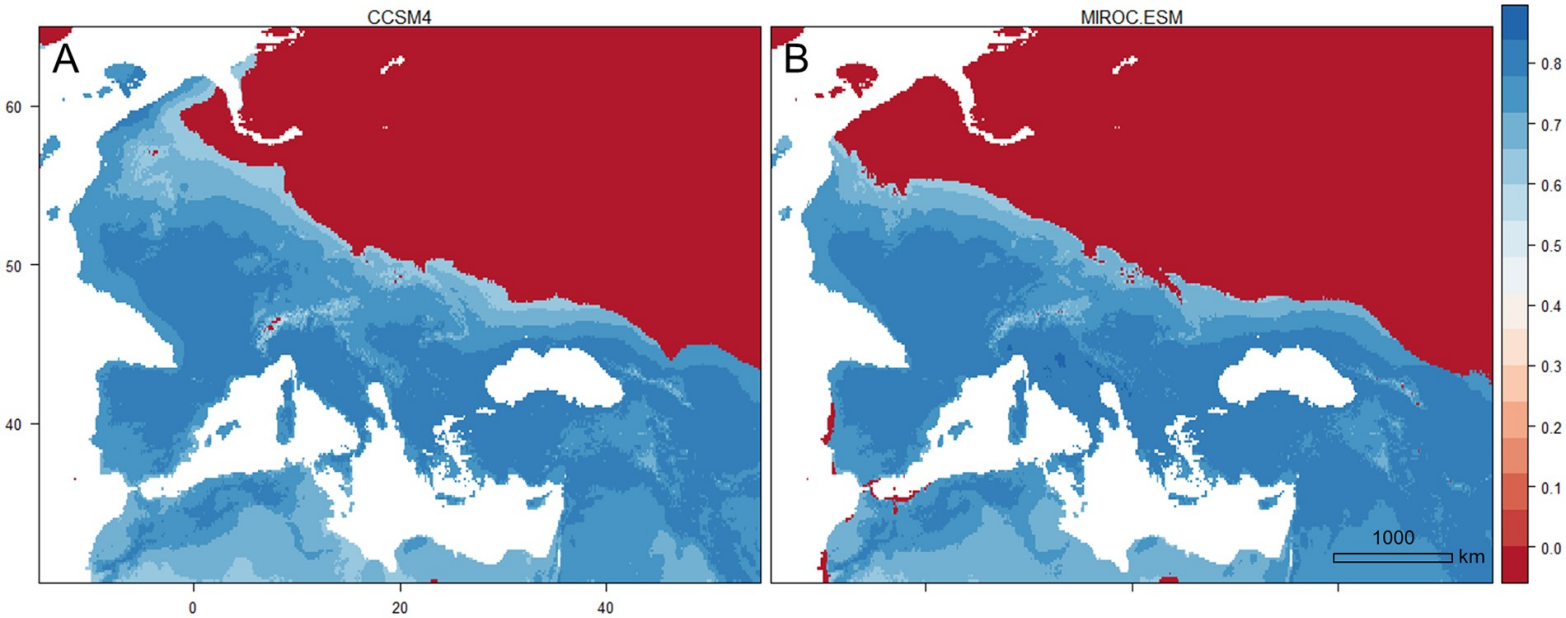
Mobility-oriented parity (MOP) analysis comparing current conditions of the calibration region for stag beetle distribution modelling with conditions during LGM. (A) Results for the CCSM4 scenario and (B) the results for MIROC-ESM. The closer the values are to 1 (dark blue) the more comparable environments are to current conditions. Areas with value 0 (dark red) are out of range for one or more environmental variables (strict extrapolation).

The MOP analyses revealed areas during LGM with environments comparable to the present-day environments in southern Europe (Fig 10). Towards the north conditions became less similar. Strict extrapolation risk was present in the northern half of Europe, which corresponded with unsuitable habitat for stag beetle in the LGM projections (under the assumption of climatic niche conservatism). Smaller areas of strict extrapolation were also present in the Alps when compared with the CCSM4 scenario (Fig 10A) and in the southern and south-western edges of Iberia when compared with the MIROC-ESM scenario (Fig 10B).

## Discussion

This study is the first phylogeographic investigation of stag beetle to incorporate both regional and fine scale sampling, and analysis of both mtDNA and nuclear genetic markers. mtDNA analysis revealed a clear spatial pattern wherein most of the European range was dominated by a single clade (lineage I). A second clade (lineage II) was found to be restricted to samples from Greece, with the differentiation of Greek samples also apparent at microsatellite loci. Microsatellite analysis further differentiated samples mainly from Central Italy that was part of lineage I on the basis of mtDNA. Nuclear data also subdivided Lineage I in two clusters. Apart from an increase in genetic clusters, differentiation among populations using nuclear markers was low to moderately high and was in agreement with the spatial structure results.

The populations in Greece did not seem to have experienced a demographic change and stayed largely isolated from populations in other regions, as the mtDNA and nuclear markers indicated. During the LGM, possible habitat is predicted to be limited to the north-western corner of Greece but was connected with a larger habitat area in southern Albania. The fact that the Greek lineage did not expand after the LGM is potentially due to the mountain ranges of northern Greece which were glaciated during the Pleistocene and could have acted as barriers isolating the populations on this part of the Balkan Peninsula [67]. Similarly, a separate lineage of *Rosalia alpina* was also found in north-western Greece by Drag et al. [68]. However, stag beetles appeared to be able to cross the Alps and Pyrenees. Fossil pollen data showed the Pindus Mountains of Greece to be a refugium of deciduous *Quercus* spp., a major host species group of stag beetle, where only a limited increase in distribution range was observed [69]. Likewise, the Greek mountains hindered post-glacial dispersal of Mediterranean oaks [70]. Thus the habitat requirements of the host species might be the underlying factor why the Greek stag beetle distribution did not shift northwards. Due to the continuous presence of suitable habitat, the population size was able to stay stable and retain its genetic diversity.

The star-like topology of lineage I is compatible with a sudden range expansion, a scenario supported by neutrality test and mismatch distribution results. The expansion appeared to be of a demographic nature as well as of a spatial nature. The BSP showed a strong increase in population size after the LGM. All diversity measures had a significant negative relation to latitude which is in line with a general pattern of increased southern refugial richness found in studies investigating post-glacial recolonisation processes in the northern hemisphere [71, 72]. During range contractions and expansions in the course of climatic oscillations, genotypes may have been lost in northern Europe, but not in populations that stayed in the southern refugia. This loss of alleles and haplotypes could have been the result of a rapid expansion of already impoverished populations from the leading edge or from single refugial populations [71]. Moreover, founder effects may have attributed to this loss of genetic diversity.

Our results suggested that, apart from Greece, another refugium was located in southern Europe. Next to Greece, Italy seemed to hold the highest genetic diversity, mitochondrial as well as nuclear. According to our palaeo-distribution model, several parts of southern Italy seemed to be a candidate refugium, going from the north in Piemonte and Genoa, through Lazio and Umbria in Central Italy, to the utmost south in Calabria. This is assuming historical climate preference to be the same as current, where winter and summer temperatures (expressed as continentality in the model) and humidity levels appeared to be important factors, which concurs with earlier findings [5, 73–75]. Italy was also one of the prime refugia during the LGM of the European white oaks [76]. The presence of admixed individuals and populations assigned to different clusters in the north of Italy based on nuclear data could further indicate the occurrence of a suture zone.

Diversity measures of the mtDNA correlated positively with longitude. This seemed to exclude northern Iberia and south-west France as refugia, although the palaeo-distribution model showed high probabilities for the presence of stag beetle in those areas. The lack of genetic richness in these areas may reflect postglacial bottlenecks. Even though in Central France higher levels of nuclear gene diversity and allelic richness were present, we have no samples from the southwest of France that could corroborate the area to have been another refugium. If this were the case, it could explain why we found cluster 4 with individuals across region ‘West’, based on nuclear DNA, which could represent another lineage. Our genetic data might also not hold enough resolving power to distinguish a secondary refuge in the southwest of France and/or northern Iberia.

Although the species is attributed limited vagility, our results showed a substantial and rapid range expansion of *L*. *cervus* in ancient times. Female stag beetles especially have the tendency to stay close to their site of emergence [16, 77, 78]. Dispersal distances are even smaller when suitable oviposition sites are abundant and nearby as in Bosco Della Fontana in Italy [78], but can become somewhat larger in suburban areas [5] and under unsuitable habitat conditions [16]. Thomaes et al. [16] found that availability of suitable dead wood for oviposition and habitat quality, defined as canopy cover, are the main drivers of dispersal in female stag beetles and thus in regulating colonisation. Our results, on the basis of the maternally inherited mtDNA, suggested that long distance dispersal across generations by female stag beetles could have occurred or that the environment at that time might have invited them to disperse more frequently than is now assumed. As the postglacial colonisation of host tree species, specifically *Quercus* species, through Europe progressed [69], suitable habitat for stag beetle must have been available, enabling females to stay, as many studies have shown. On the other hand, nesting sites might have been distributed in a scattered manner, which could have induced dispersal. When numbers of larvae also increased locally, this could have encouraged the females to look for other nesting sites, as they seem not to lay their eggs where other larvae are already present [79].

While mtDNA remains the classical phylogeographical marker it may lack the power to detect population structure on fine spatio-temporal scales, particularly in cases where individuals are derived from star-shaped phylogenies. Microsatellites, owing to their high levels of variability, may confer greater fine scale resolution. Founder effects and genetic bottlenecks during colonisation would have reduced local population genetic diversity and increased genetic differentiation among populations, especially when rates of population growth remained small after colonisation [80]. However, levels of nuclear genetic diversity in northern and western populations were not extremely low and no indication of inbreeding was found. Here microsatellites revealed often low to moderate F_ST_ values within clusters and the lack of a significant signal of IBD when Greek samples were excluded. This might suggest gene flow among populations with distances exceeding the assumed home range between them to be possible. Nonetheless, the F_ST_ values may represent the historical rather than the present pattern, due to shared alleles from an ancestral population. Furthermore, population differentiation was often significant, even among nearby populations (e.g. the Belgian populations). Finer scale resolution of the drivers of structure here will require further sampling.

As for many other species hot spots of genetic diversity for *L*. *cervus* seemed to lie in south-eastern Europe where they resided during the LGM. The identification of such regions, especially in Greece and Italy, could help conservation managers to prioritise actions to preserve the current level of genetic diversity of the species. The study delivered new knowledge on the colonisation history of *L*. *cervus* in Europe after the LGM, as the data demonstrated that a single clade successfully colonised most of Europe. However, further research is needed on a more local scale to evaluate the drivers of fine scale structure among populations under current conditions and if effective population sizes are high enough to counter the negative effects of isolation.

## Supporting information

S1 Fig Maximum-likelihood phylogeny tree, calculated with RAxML for *Lucanus cervus COI* data, using *L*. *ibericus* as an outgroup species. Only bootstrap values greater than 70% are shown (100 replicates). The scale bar corresponds to the mean number of amino acid substitutions per site on the respective branch. Lineages I and II are indicated with blue and pink, respectively.
(TIF)

S2 Fig Spatial clustering results based on microsatellite genotypes of *Lucanus cervus*. (A) The BAPS results showing four clusters, (B-D) STRUCTURE results with (B) K = 2 main clusters involving all samples, and with (C) K = 2 subclusters and (D) K = 4 subclusters after excluding Greek samples assigned to one cluster in (B; indicated in pink). The estimated probabilities of assignment to each cluster (indicated in different colours) are shown on the y-axes.
(TIF)

S3 Fig ΔK calculated for each K from 1 to 20 using the Evanno method in STRUCTURE HARVESTER. This is based on STRUCTURE results (A) using all samples and (B) after excluding the Greek samples.
(TIF)

S4 Fig The final set of occurrence data of *Lucanus cervus* after thinning indicated with red x’s. The insert shows part of Southern England where grey crosses are occurrence sites before the final thinning step using the nearest neighbour distance of 5 km.
(TIF)

S1 Table Sample information of the collected stag beetles including localities, coordinates, year of collection and accession numbers of the studied *COI* sequences. (PDF)

S2 Table Microsatellite multiplex and simplex primer concentrations, fluorescent labels and annealing temperatures for *Lucanus cervus*. (PDF)

S3 Table Population pairwise F_ST_ values for mtDNA dataset of *Lucanus cervus*. Significant values (p < 0.05) are indicated in bold.
(PDF)

S4 Table Population pairwise F_ST_ values for microsatellite genotypes of *Lucanus cervus*. Significant values (p < 0.05) are indicated in bold.
(PDF)

S5 Table *Lucanus cervus* microsatellite data used in the analyses. (XLSX)
