# Supplementary figures and images for: Phylogeographic structure and ecological niche modelling reveal signals of isolation and postglacial colonisation in the European stag beetle
Source: PLoS One. 2019 Apr 25;14(4):e0215860. doi: 10.1371/journal.pone.0215860 (PMC6483211; doi:10.1371/journal.pone.0215860)

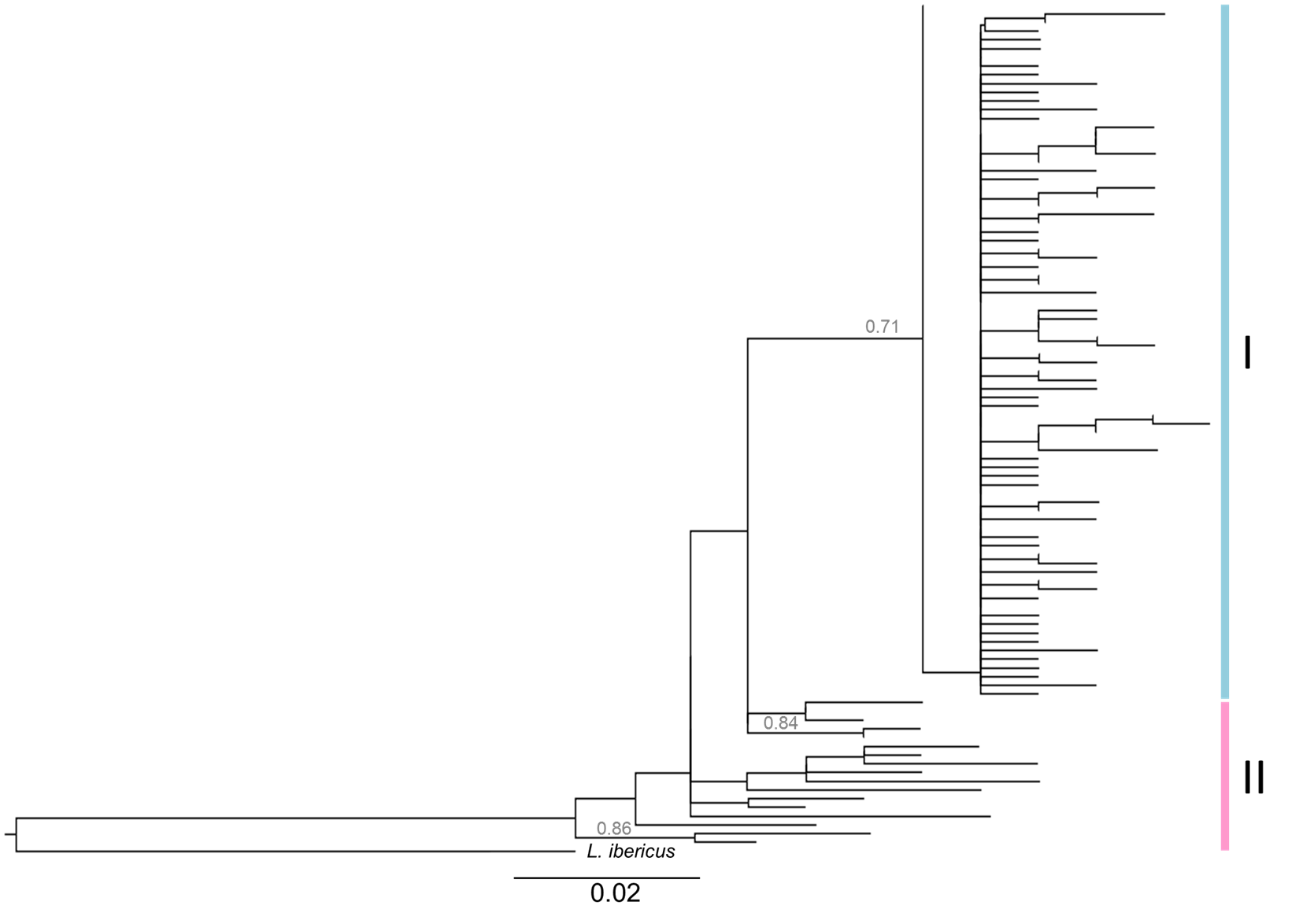

Supplement: S1 Fig — Only bootstrap values greater than 70% are shown (100 replicates). The scale bar corresponds to the mean number of amino acid substitutions per site on the respective branch. Lineages I and II are indicated with blue and pink, respectively. (TIF) [file pone.0215860.s001.tif]

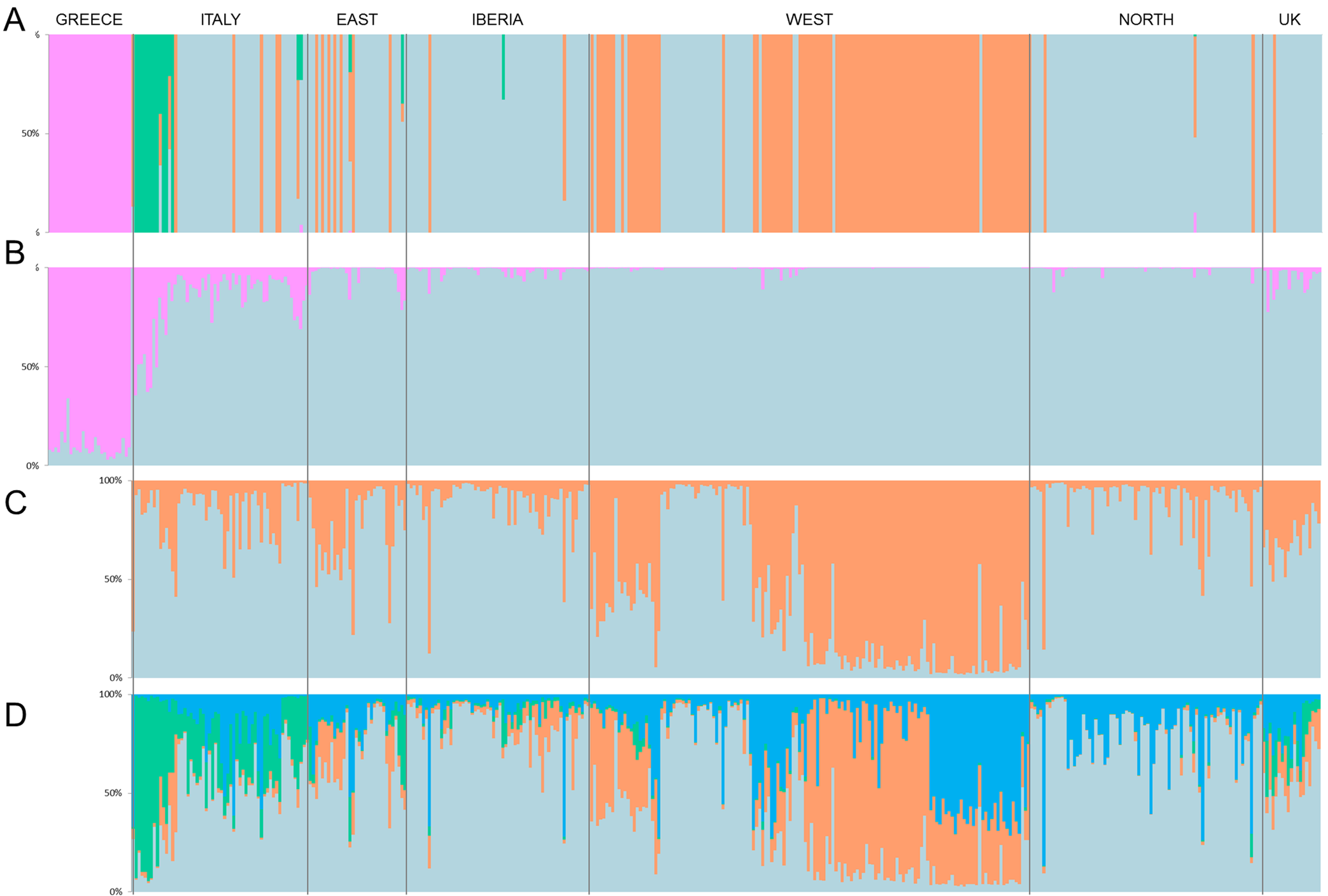

Supplement: S2 Fig — (A) The BAPS results showing four clusters, (B-D) STRUCTURE results with (B) K = 2 main clusters involving all samples, and with (C) K = 2 subclusters and (D) K = 4 subclusters after excluding Greek samples assigned to one cluster in (B; indicated in pink). The estimated probabilities of assignment to each cluster (indicated in different colours) are shown on the y-axes. (TIF) [file pone.0215860.s002.tif]

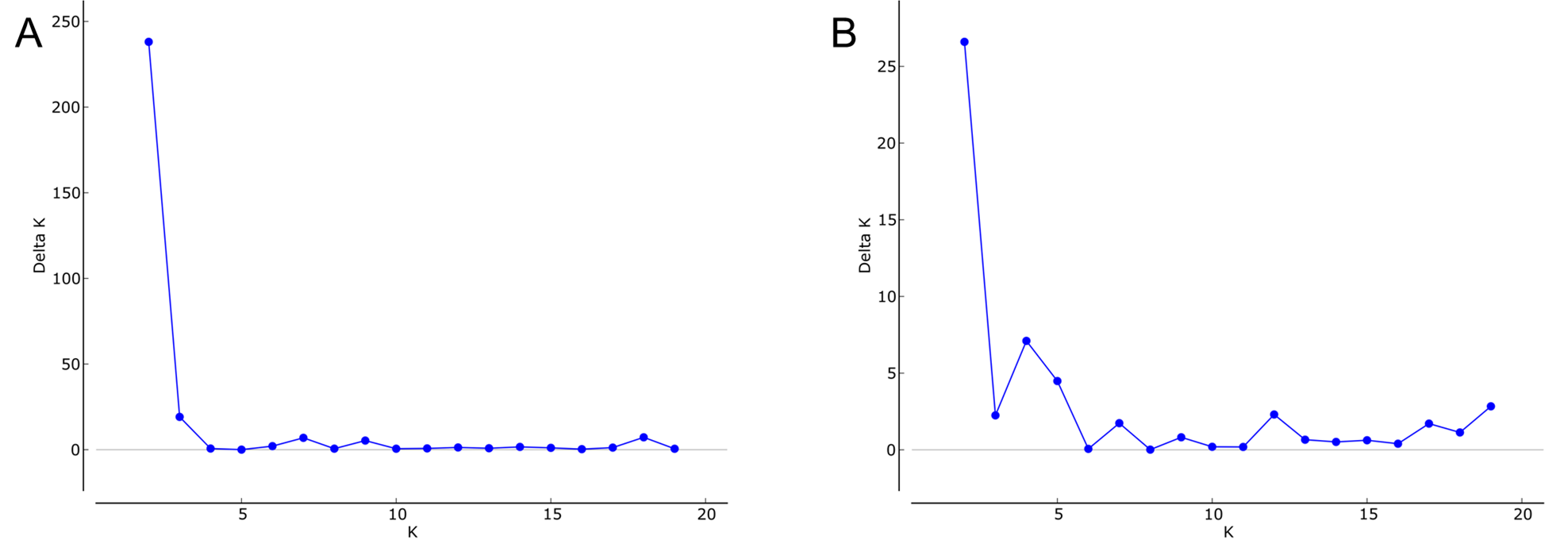

Supplement: S3 Fig — This is based on STRUCTURE results (A) using all samples and (B) after excluding the Greek samples. (TIF) [file pone.0215860.s003.tif]

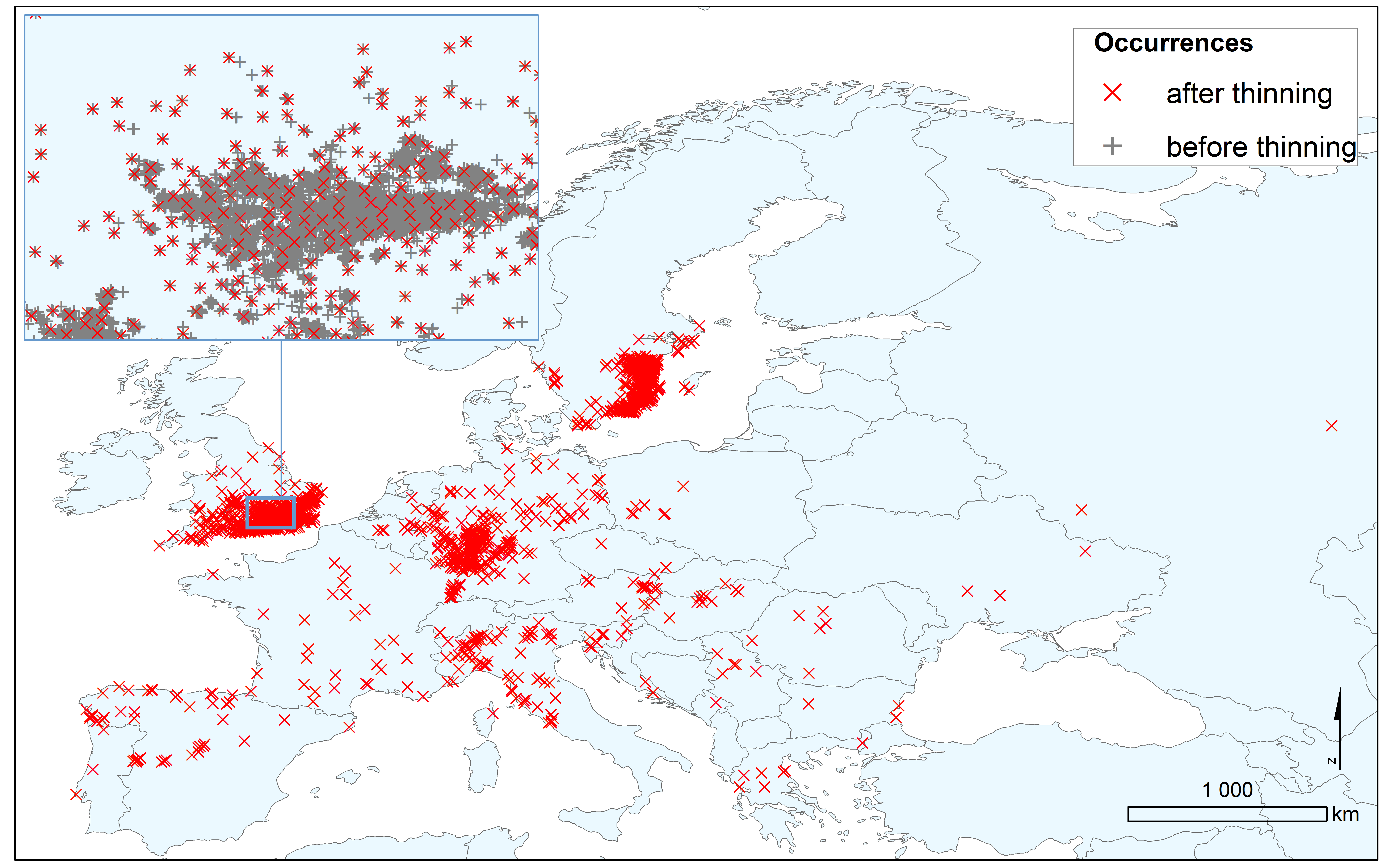

Supplement: S4 Fig — The insert shows part of Southern England where grey crosses are occurrence sites before the final thinning step using the nearest neighbour distance of 5 km. (TIF) [file pone.0215860.s004.tif]
