# Supplementary material for: Phylogeographic structure and ecological niche modelling reveal signals of isolation and postglacial colonisation in the European stag beetle
Source: PLoS One. 2019 Apr 25;14(4):e0215860. doi: 10.1371/journal.pone.0215860 (PMC6483211; doi:10.1371/journal.pone.0215860)
Supplement: S1 Table — (PDF) [file pone.0215860.s005.pdf]

S1 Table. **Sample information of the collected stag beetles including localities, coordinates, year of collection and accession numbers of the studied *COI* sequences.**

Region	Country	Locality	Loc. No.	Lon	Lat	N_coi	N_ssr	N in common	year	Genbank acc. No.
West	The Netherlands	Jabeek	1	5.9400	50.9806	1	1	1	2009	MK783447
		Onderbanken	2	5.9653	50.9697	0	1	0	2009	
	Belgium	Sint-Genesius-Rode	3	4.3319	50.7360	2	10	2	2009-2010	KF737072[a], MK783322
		Watermaal-Bosvoorde	4	4.4140	50.7967	0	30	0	2005	
		Overijse	5	4.5272	50.7723	2	30	2	2008-2009	KF737071[a], MK783323
	France	Bussiere	6	4.3803	45.5869	18	0	0	2001-2005	MK783336 - MK783340, MK783342 - MK783354
		Naucelle	7	2.3424	44.1984	1	1	1	2010	MK783335
		Basses-Pyrenees	8	-1.4803	43.4807	1	0	0		FJ606555[b]
		La Caulie	9	2.2886	43.6154	2	4	2	2009	MK783327, MK783357
		Lanouaille	10	1.1393	45.3918	3	5	3	2010	KF737078[a], MK783328, MK783329
		Clermont-le-Fort	11	1.4318	43.4581	3	4	3	2009-2010	KF737088[a], MK783341, MK783356
		Lurais	12	0.9513	46.7051	5	5	5	2010	MK783330 - MK7833334
		Chateauneuf-sur-Loire	13	2.2217	47.8647	1	1	1	2009	MK783326
		Montbouy	14	2.8211	47.8644	1	2	1	2009	KF737092[a]
	Germany	Kranenburg	15	6.0128	51.7514	1	1	1	2009	MK783448
		Alf	16	7.1167	50.0500	6	5	3	2003-2015	MK783355, MK783358, MK783359, MK783368, MK783371, MK783374
		Schaidt	17	8.1000	49.0167	0	1	0	2007	
		Forst	18	8.5833	49.1500	5	10	5	2009	MK783360, MK783365 - MK783367, MK783372
		Kronau	19	8.6333	49.2167	5	15	5	2009	MK783361, MK783362, MK783369, MK783370, MK783373
		Rettigheim	20	8.7000	49.2333	2	4	2	2009	MK783376, MK783377
		Tairnbach	21	8.7500	49.2500	2	13	2	2009	MK783363, MK783364
		Melsbach	22	7.4667	50.4833	1	1	1	2009	MK783375
Greece	Greece	Ioannina	23	20.8401	39.6745	1	1	1	2010	MK783378
		Dadia	24	26.2257	41.1280	4	4	4	2010	MK783392 - MK783395
		Neraida	25	21.7903	39.1408	5	5	5	2009	MK783380, MK783383, MK783390, MK783391, MK783396
		Zalongo	26	20.6667	39.1333	1	1	1	2010	MK783389

Region	Country	Locality	Loc. No.	Lon	Lat	N_coi	N_ssr	N in common	year	Genbank acc. No.
		Nessonas	27	22.6539	39.8083	3	4	3	2009	KF737079[a], KF737083[a], MK783379
		Stomio	28	22.7333	39.8667	0	1	1	2009	
		Vlahava	29	21.6633	39.7623	8	12	8	2009	KF737082[a], MK783381, MK783382, MK783384 - MK783388
East	Hungary	Kesztölc	30	18.8346	47.7016	2	3	2	2009	KF737081[a], MK783400
		Nagymaros	31	18.9587	47.7867	1	1	1	2007	MK783401
		Vac	32	19.1377	47.7754	3	3	3	2004	MK783398, MK783399, MK783403
		Budapest	33	18.9660	47.5027	1	1	1	2005	MK783402
		Isaszeg	34	19.3991	47.5516	1	1	1	2009	MK783397
		Kőszeg	35	16.5522	47.7754	0	1	0	2009	
	Montenegro	Mojkovac	36	19.5825	42.9602	1	0	0		KF737133[a]
	Romania	Bistrița	37	24.4507	47.1024	7	0	0	2001-2005	KF737087[a], MK783462, MK783463, MK783474 - MK783477
		Craiova	38	23.8056	44.2978	1	2	1	1988	MK783471
		Târnăveni	39	24.2969	46.3267	2	5	2	2006-2007	MK783472, MK783473
		Târgu Mureș	40	24.5672	46.5428	1	1	1	2007	MK783461
		Timisoara	41	21.2300	45.7597	7	0	0	2001-2005	MK783464 - MK783470
	Serbia	Andreljev	42	19.6476	45.1737	2	2	2	2009	MK783483, MK783484
	Slovenia	Podsmreka	43	14.4295	46.0390	1	1	1	2009	MK783487
		Ljubljana	44	14.5060	46.0514	0	1	0	2010	
		Lokavec	45	13.8792	45.9347	1	1	1	2009	MK783485
		Pivka	46	14.1960	45.6825	7	9	7	2009	MK783486, MK783490 - MK783495
		Poljčane	47	15.5647	46.3033	2	2	2	2009	MK783488, MK783489
Italy	Italy	Marmirolo	48	10.7422	45.2007	20	34	20	2009-2015-2017	KU139611* [c], MK783407, MK783408, MK783422 - MK783424, MK783426 - MK783430, MK783436 - MK783442, MK783444 - MK783446
		Manziana	49	12.1253	42.1311	2	0	0		KU139612[c], KU139613[c]
		Castel Guiliano	50	12.1259	42.0536	4	0	0		KU139638[c], KU139640[c], KU139610[c], KU139606[c]
		Sondrio	51	9.8711	46.1688	10	0	0		KU139620[c], KU139621[c], KU139622[c], KU139623[c],

Region	Country	Locality	Loc. No.	Lon	Lat	N_coi	N_ssr	N in common	year	Genbank acc. No.
										KU139624[c], KU139625[c], KU139626[c], KU139627[c], KU139629[c], KU139630[c]
		Orrido di Botri	52	10.6246	44.0862	4	4	4	2009	KU139632* [c], KU139634* [c], KU139635 [c], KU139633* [c], MK783431, MK783433, MK783435
		Biforco	53	11.6000	44.0667	2	2	2	2009	MK783405, MK783406
		Roccalbegna	54	11.5039	42.7491	2	0	0	2009	KU139614[c], KU139615* [c], MK783421
		Bernate	55	8.7353	45.7798	10	9	9	2009	KU139619[c], KF737084[a], KF737080[a], KF737085▲[a], MK783404, MK783425, MK783432, MK783434, MK783443
		Bodio Lomnago	56	8.7541	45.7888	1	0	0		KU139618[c]
		Varese	57	497208.2	-378306	1	0	0		KU139637[c]
		Acquapendente	58	12.1048	42.4170	11	8	8	2009	KU139607* [c], KU139617* [c], KU139628[c], KU139636* [c], MK783409 - MK783419
		Viterbo	59	12.1095	42.4226	1	0	0	2009	KU139631* [c], MK783420
		Ronciglione	60	12.2147	42.2885	1	0	0		KU139616[c]
North	Czech Republic	Roztoky u Prahy	61	14.3928	50.1622	0	1	0	2007	
		Lednice	62	16.8036	48.7979	3	4	3	2009	KF737093[a], MK783324, MK783325
		Buchlovice	63	17.3333	49.0833	0	1	0	2008	
	Poland	Postolin	64	17.2367	51.4760	1	1	1	2010	MK783450
		Milicz	65	17.2868	51.5177	3	5	3	2015	MK783452, MK783453, MK783455
		Janikow	66	18.1133	52.7538	2	5	2	2010	MK783456, MK783457
		Pnewkow	67	15.8567	51.5231	5	37	5	2010	MK783449, MK783451, MK783454, MK783458, MK783459
		Joolkow	68	15.9618	51.8612	0	4	0	2010	
	Ukraine	Vasischeve	69	36.3258	49.8269	1	2	1	2007	KF737090[a]
		Migiiska-Dilanka	70	30.9804	48.0100	0	1	0	2009	
		Berezovka	71	32;4589	47.8128	4	5	4	2009	MK783676 - MK783679
	Russia	Yandyichi	72	47.4995	55.5054	1	1	1	2009	MK783479
		Kursk	73	36.1720	51.6862	3	6	3	1990	MK783480 - MK783482

Region	Country	Locality	Loc. No.	Lon	Lat	N_coi	N_ssr	N in common	year	Genbank acc. No.
		Bessonovka	74	48.2540	50.7783	1	1	1	2009	MK783478
	Sweden	Ronneby	75	15.2761	56.2100	1	1	1	2009	MK783520
		Linköping	76	15.6254	58.4158	1	1	1	2009	MK783521
Iberia	Portugal	Gerês	77	-8.1667	41.7334	0	1	0	2008	
		Sintra	78	-9.3974	38.7959	2	3	2	2010	KF737086[a], MK783460
		Quinta	79	-8.5691	39.9302	0	1	0	2004	
	Spain	El Caliero	80	-6.1511	43.4822	0	1	0	2009	
		Illas	81	-5.9761	43.4992	2	4	2	2009	MK783509, MK783513
		La Candaliega	82	-5.9603	43.5604	0	1	0	2009	
		La Laguna	83	-5.9761	43.4992	0	6	0	2015	
		Avilés	84	-5.9220	43.5554	0	3	0	2009	
		Las Arenas de Cabrales	85	-4.8150	43.3040	2	2	2	2009	KF737076[a], MK783504
		Garganta de Navamediana	86	-5.4141	40.3174	1	1	1	2009	MK783496
		Dehesa de la Lastra del Cano	87	-5.4070	40.3540	0	1	0	2009	
		Navalguijo	88	-5.5222	40.2585	0	1	1	2009	
		Zapardiel de la Ribera	89	-5.3286	40.3558	1	1	1	2009	MK783503
		Guriezo	90	-3.3255	43.3544	0	1	0	2009	
		Castro Urdiales	91	-3.2196	43.3828	0	2	0	2009	
		Cervo	92	-7.4477	43.6821	1	1	1	2009	MK783514
		Ribadeo	93	-7.0430	43.5374	0	1	0	2009	
		El Ferrol	94	-7.0297	43.5144	0	1	0	2009	
		A Proba do Brollón	95	-7.3924	-42.5570	1	1	1	2009	MK783519
		Manzanares el Real	96	-3.8649	40.7271	2	2	2	2009	MK783506, MK783507
		Rascafría	97	-3.8794	40.9038	1	1	1	2009	MK783512
		Soto del Real	98	-3.8156	40.7438	1	1	1	2009	MK783505
		Buitrago de Lozoya	99	-3.6367	40.9937	1	1	1	2009	MK783515
		Horcajo de la Sierra	100	-3.5856	41.0672	2	0	0	2015	MK783511, MK783516
		El Escorial	101	-4.1585	40.5806	0	2	0	2009	
		Villaescusa de las Torres	102	-4.2544	42.7595	1	1	1	2009	MK783517
		Navasfrias	103	-6.8201	40.2964	0	1	0	2009	
		La Genestosa	104	-6.7863	40.3504	2	3	2	2009	MK783497, MK783510
		Cassillas de Flores	105	-6.7542	40.3821	1	2	1	2009	MK783500
		Dehesa de El Payo	106	-6.6815	40.2758	2	2	2	2009	MK783501, MK783502
		Villasrubias	107	-6.6403	40.3381	1	2	1	2009	MK783518

Region	Country	Locality	Loc. No.	Lon	Lat	N_coi	N_ssr	N in common	year	Genbank acc. No.
		Dehesa-de-Robleda	108	-6.6085	40.3851	3	4	3	2009	KF737094[a], MK783498, MK783499
		Martiago	109	-6.4900	40.4524	0	1	0	2009	
		Agallas	110	-6.4421	40.4487	0	1	0	2009	
		Nuévalos	111	-1.7898	41.2120	1	1	1	2009	MK783508
UK	United Kingdom	Berkshire	112	-1.2814	51.4660	20	0	0	2001-2005	MK783541 - MK783544, MK783546 - MK783555, MK783557 - MK783562
		Essex	113	0.6674	51.7659	20	0	0	2001-2005	MK783563 - MK783566, MK783568 - MK783577, MK783579 - MK783584
		Colchester	114	0.9012	51.8898	3	13	3	2005-2006	MK783522, MK783585, MK783586
		Hampshire	115	-1.2168	51.0895	20	0	0	2001-2005	MK783524 - MK783533, MK783535 - MK783540, MK783672 - MK783675
		Kent	116	0.6674	51.7659	20	0	0	2001-2005	KF737089[a], MK783616 - MK783620, MK783622 - MK783631, MK783633 - MK783636
		London	117	-0.1262	51.5002	16	0	0	2001-2005	MK783655 - MK783664, MK783666 - MK783671
		Suffolk	118	0.8443	51.2601	16	0	0	2001-2005	MK783637 - MK783642, MK783644 - MK783653
		Copdock	119	1.0674	52.0289	4	6	4	2009	KF737077[a], MK783600, MK783611, MK783621
		Surrey	120	-0.4673	51.2623	20	0	0	2001-2005	MK783523, MK783534, MK783545, MK783556, MK783567, MK783578, MK783587 - MK783596, MK783632, MK783643, MK783654, MK783665
		Sussex	121	-0.2093	50.9663	18	0	0	2001-2005	KF737091[a], MK783597 - MK783599, MK783601 - MK783610, MK783612 - MK783615

Loc. No., locality number as indicated in Fig 1; N_coi, number of samples with sequences of COI; N_ssr:,number of samples with microsatellite genotypes; N in common, number samples with COI sequence and microsatellite genotypes available.

* sequenced again in this study.

▲ another individual from Bernate (Italy) with the same haplotype as KF737085 was included; it does not have a separate accession number.

[a] Cox K, Thomaes A, Antonini G, Zilioli M, De Gelas K, Harvey D, et al. Testing the performance of a fragment of the COI gene to identify western Palaearctic stag beetle species (Coleoptera, Lucanidae). ZooKeys. 2013;365(0):105-26. doi: 10.3897/zookeys.365.5526.
[b] Lin C-P, Huang J-P, Lee Y-H, Chen M-Y. Phylogenetic position of a threatened stag beetle, *Lucanus datunensis* (Coleoptera: Lucanidae) in Taiwan and implications for conservation. Conserv Genet. 2011;12(1):337-41. doi: 10.1007/s10592-009-9996-8.
[c] Solano E, Thomaes A, Cox K, Carpaneto GM, Cortellessa S, Baviera C, et al. When morphological identification meets genetic data: the case of *Lucanus cervus* and *L. tetraodon* (Coleoptera, Lucanidae). J Zool Syst Evol Res. 2016:n/a-n/a. doi: 10.1111/jzs.12124.
