# Supplementary material for: Phylogeographic structure and ecological niche modelling reveal signals of isolation and postglacial colonisation in the European stag beetle
Source: PLoS One. 2019 Apr 25;14(4):e0215860. doi: 10.1371/journal.pone.0215860 (PMC6483211; doi:10.1371/journal.pone.0215860)
Supplement: S2 Table — (PDF) [file pone.0215860.s006.pdf]

S2 Table. Microsatellite multiplex and simplex primer concentrations, fluorescent labels and annealing temperatures for *Lucanus cervus*.

Locus	Multiplex set no.	Fluorescent label	T_a	voMume
Lcerv-1	1	fam	47	0.2 µM
Lcerv-4	1	ned	47	0.2 µM
Lcerv-6	1	pet	47	0.2 µM
Lcerv-21	1	vic	47	0.2 µM
Lcerv-3	2	fam	46	0.2 µM
Lcerv-9	2	ned	46	0.2 µM
Lcerv-25	2	pet	46	0.2 µM
Lcerv-28	2	vic	46	0.1 µM
Lcerv-8	3	vic	50	0.05 µM
Lcerv-30	3	ned	50	0.05 µM
Lcerv-31	3	fam	50	0.2 µM
Lcerv-16	4	vic	54	0.2 µM
Lcerv-17	4	fam	54	0.2 µM
Lcerv-20	4	fam	54	0.05 µM
Lcerv-36	4	pet	54	0.4 µM
Lcerv-29	4	ned	54	0.2 µM
Lcerv-7	simplex	pet	50	0.1 µM

T_a: annealing temperature in °C.
