# Supplementary material for: Phylogeographic structure and ecological niche modelling reveal signals of isolation and postglacial colonisation in the European stag beetle
Source: PLoS One. 2019 Apr 25;14(4):e0215860. doi: 10.1371/journal.pone.0215860 (PMC6483211; doi:10.1371/journal.pone.0215860)
Supplement: S3 Table — Significant values (p < 0.05) are indicated in bold. (PDF) [file pone.0215860.s007.pdf]

S3 Table. Population pairwise F_{ST} values for mtDNA dataset of *Lucanus cervus*. Significant values ($p < 0.05$) are indicated in bold.

	**Bussière**	**Lurais**	**Alf**	**Forst**	**Kronau**	**Pnewkow**	**Neraida**	**Vlahava**	**Bistrita**	**Timisoara**	**Pivka**	**Aquapend ente**	**Bernate**	**Marmirolo**	**Sondrio**	**Berkshire**	**Essex**	**Hampshir e**	**Kent**	**London**	**Suffolk**	**Surrey**	**Sussex**
**Bussière**	-																						
**Lurais**	0.075	-																					
**Alf**	0.075	-0.004	-																				
**Forst**	-0.054	0.000	-0.034	-																			
**Kronau**	0.026	0.000	-0.015	0.000	-																		
**Pnewkow**	0.075	0.000	-0.004	0.000	-0.154	-																	
**Neraida**	**0.797**	**0.628**	**0.642**	**0.656**	**0.638**	**0.621**	-																
**Vlahava**	**0.580**	**0.380**	**0.390**	**0.391**	**0.378**	**0.373**	**0.132**	-															
**Bistrita**	**0.719**	**0.608**	**0.593**	**0.691**	**0.647**	**0.608**	**0.697**	**0.514**	-														
**Timisoara**	**0.736**	**0.629**	**0.614**	**0.710**	**0.667**	**0.629**	**0.701**	**0.521**	-0.143	-													
**Pivka**	**0.326**	0.184	0.185	0.198	0.199	0.184	**0.655**	**0.435**	**0.591**	**0.609**	-												
**Aquapendente**	0.037	-0.036	-0.018	-0.089	-0.060	-0.036	**0.673**	**0.457**	**0.518**	**0.540**	**0.152**	-											
**Bernate**	**0.067**	-0.014	-0.023	-0.082	-0.034	-0.014	**0.650**	**0.426**	**0.533**	**0.554**	0.134	0.013	-										
**Marmirolo**	**0.037**	0.003	0.001	-0.081	-0.034	0.003	**0.771**	**0.571**	**0.612**	**0.632**	**0.231**	0.021	**0.044**	-									
**Sondrio**	**0.102**	-0.001	0.017	-0.063	-0.015	-0.025	**0.624**	**0.431**	**0.488**	**0.509**	0.134	**0.036**	-0.017	**0.077**	-								
**Berkshire**	**0.040**	0.092	0.087	-0.105	-0.040	0.047	**0.827**	**0.620**	**0.762**	**0.777**	**0.373**	**0.036**	**0.072**	0.024	**0.115**	-							
**Essex**	**0.352**	**0.267**	**0.273**	0.261	0.264	0.240	**0.756**	**0.582**	**0.616**	**0.632**	**0.367**	**0.258**	**0.270**	**0.306**	**0.259**	**0.377**	-						
**Hampshire**	**0.051**	0.016	0.030	-0.065	-0.071	-0.028	**0.771**	**0.574**	**0.618**	**0.638**	**0.240**	0.033	0.045	**0.042**	0.017	0.010	**0.313**	-					
**Kent**	**0.163**	0.096	0.107	0.057	0.078	0.096	**0.751**	**0.565**	**0.582**	**0.601**	**0.258**	**0.098**	**0.121**	**0.138**	**0.116**	**0.172**	0.039	**0.146**	-				
**London**	0.062	0.123	0.106	-0.049	0.071	0.123	**0.810**	**0.588**	**0.766**	**0.781**	**0.375**	**0.035**	**0.072**	0.029	**0.107**	0.028	**0.368**	0.008	**0.168**	-			
**Suffolk**	**0.158**	0.111	0.115	0.063	0.090	0.000	**0.765**	**0.560**	**0.652**	**0.671**	**0.296**	**0.090**	**0.112**	**0.114**	**0.100**	**0.165**	**0.193**	**0.124**	**0.094**	**0.173**	-		
**Surrey**	**0.083**	0.137	0.125	-0.024	0.082	0.137	**0.831**	**0.625**	**0.773**	**0.787**	**0.395**	**0.057**	**0.095**	**0.049**	**0.119**	0.053	**0.389**	**0.065**	**0.188**	0.086	**0.192**	-	
**Sussex**	**0.061**	0.079	0.018	-0.058	0.027	0.079	**0.806**	**0.590**	**0.727**	**0.744**	**0.336**	**0.039**	0.046	**0.037**	**0.106**	**0.036**	**0.358**	**0.052**	**0.167**	0.059	**0.161**	**0.079**	-
