# Supplementary material for: Phylogeographic structure and ecological niche modelling reveal signals of isolation and postglacial colonisation in the European stag beetle
Source: PLoS One. 2019 Apr 25;14(4):e0215860. doi: 10.1371/journal.pone.0215860 (PMC6483211; doi:10.1371/journal.pone.0215860)
Supplement: S4 Table — Significant values (p < 0.05) are indicated in bold. (PDF) [file pone.0215860.s008.pdf]

S4 Table. Population pairwise F_{ST} values for microsatellite genotypes of *Lucanus cervus*. Significant values ($p < 0.05$) are indicated in bold.

	Overijse	Sint-Genesius-Rode	Watermaal-Bosvoorde	Lanouaille	Lurais	Alf	Forst	Kronau	Tairnbach	Vlahava	Neraida	Viterbo	Marmirolo	Bernate	Milicz	Janikow	Pnewkow	Tarnaveni	Kursk	Pivka	La Laguna	Berezovka	Colchester	Copdock
**Overijse**	-																							
**Sint-Genesius-Rode**	**0.059**	-																						
**Watermaal-Bosvoorde**	**0.053**	**0.084**	-																					
**Lanouaille**	**0.061**	**0.115**	**0.067**	-																				
**Lurais**	0.050	**0.071**	0.043	0.042	-																			
**Alf**	**0.063**	**0.088**	**0.066**	0.055	0.047	-																		
**Forst**	**0.110**	**0.123**	**0.106**	**0.091**	0.070	0.037	-																	
**Kronau**	**0.092**	**0.098**	**0.081**	**0.078**	0.051	0.031	0.029	-																
**Tairnbach**	**0.063**	**0.091**	**0.037**	0.052	0.027	0.042	**0.065**	**0.043**	-															
**Vlahava**	**0.136**	**0.176**	**0.130**	**0.109**	**0.108**	**0.106**	**0.123**	**0.113**	**0.094**	-														
**Neraida**	**0.149**	**0.171**	**0.134**	**0.126**	**0.116**	**0.136**	**0.166**	**0.146**	**0.124**	**0.056**	-													
**Viterbo**	**0.115**	**0.140**	**0.122**	**0.096**	**0.086**	**0.098**	**0.096**	**0.103**	**0.102**	**0.093**	**0.128**	-												
**Marmirolo**	0.067	0.100	0.066	0.058	0.047	0.030	0.039	0.019	0.035	0.096	**0.136**	0.083	-											
**Bernate**	0.106	0.120	0.121	0.106	0.070	0.067	0.064	0.045	0.077	0.094	**0.138**	0.086	0.048	-										
**Milicz**	**0.102**	**0.129**	**0.108**	**0.097**	0.080	0.063	0.071	0.036	0.056	**0.103**	**0.150**	**0.127**	0.035	**0.062**	-									
**Janikow**	**0.120**	**0.147**	**0.108**	**0.080**	0.071	0.081	0.075	0.039	**0.073**	**0.124**	**0.158**	**0.111**	0.042	**0.074**	0.060	-								
**Pnewkow**	**0.132**	**0.134**	**0.093**	**0.103**	**0.067**	0.058	**0.048**	0.025	**0.055**	**0.126**	**0.164**	**0.124**	**0.041**	**0.061**	**0.062**	0.048	-							
**Tarnaveni**	**0.102**	**0.120**	**0.099**	**0.096**	0.071	0.057	0.075	0.041	**0.074**	**0.124**	**0.155**	**0.123**	0.049	0.061	0.061	0.056	0.043	-						
**Kursk**	**0.115**	**0.141**	**0.101**	**0.092**	0.087	0.060	0.074	0.045	**0.070**	**0.123**	**0.162**	**0.129**	0.041	**0.074**	0.053	0.056	0.034	0.032	-					
**Pivka**	**0.072**	**0.095**	**0.061**	**0.069**	0.039	**0.059**	**0.064**	**0.048**	0.043	**0.094**	**0.120**	**0.098**	**0.049**	**0.051**	0.057	0.061	**0.060**	0.065	0.077	-				
**La Laguna**	**0.094**	**0.085**	**0.120**	**0.110**	**0.091**	0.053	0.063	0.045	**0.089**	**0.133**	**0.166**	**0.119**	**0.060**	**0.059**	0.078	**0.098**	**0.097**	**0.097**	**0.104**	**0.080**	-			
**Berezovka**	**0.178**	**0.207**	**0.198**	**0.155**	**0.148**	**0.121**	**0.137**	**0.108**	**0.160**	**0.168**	**0.202**	**0.174**	**0.101**	**0.097**	**0.126**	**0.129**	**0.121**	0.072	0.087	**0.131**	**0.159**	-		
**Colchester**	**0.060**	**0.094**	**0.071**	**0.074**	0.064	**0.053**	**0.071**	**0.046**	**0.050**	**0.099**	**0.131**	**0.095**	**0.037**	**0.053**	0.037	**0.073**	**0.079**	0.069	**0.061**	**0.046**	0.056	**0.121**	-	
**Copdock**	**0.079**	**0.104**	**0.096**	**0.093**	0.075	**0.059**	**0.079**	0.051	**0.087**	**0.115**	**0.138**	**0.106**	**0.051**	**0.080**	0.076	0.067	**0.092**	0.072	**0.085**	**0.075**	0.068	**0.116**	0.059	-
